# Dissolving Cellulose in 1,2,3-Triazolium- and Imidazolium-Based Ionic Liquids with Aromatic Anions

**DOI:** 10.3390/molecules25153539

**Published:** 2020-08-02

**Authors:** Martin Brehm, Julian Radicke, Martin Pulst, Farzaneh Shaabani, Daniel Sebastiani, Jörg Kressler

**Affiliations:** Institut für Chemie, Martin-Luther-Universität Halle–Wittenberg, von-Danckelmann-Platz 4, D-06120 Halle, Germany; julian.radicke@chemie.uni-halle.de (J.R.); martin.pulst@chemie.uni-halle.de (M.P.); farzanehautumn@gmail.com (F.S.); daniel.sebastiani@chemie.uni-halle.de (D.S.); joerg.kressler@chemie.uni-halle.de (J.K.)

**Keywords:** ionic liquids, cellulose, triazolium, solvent, carbenes, molecular dynamics, enthalpy of solvation, solubility prediction

## Abstract

We present 1,2,3-triazolium- and imidazolium-based ionic liquids (ILs) with aromatic anions as a new class of cellulose solvents. The two anions in our study, benzoate and salicylate, possess a lower basicity when compared to acetate and therefore should lead to a lower amount of *N*-heterocyclic carbenes (NHCs) in the ILs. We characterize their physicochemical properties and find that all of them are liquids at room temperature. By applying force field molecular dynamics (MD) simulations, we investigate the structure and dynamics of the liquids and find strong and long-lived hydrogen bonds, as well as significant π–π stacking between the aromatic anion and cation. Our ILs dissolve up to 8.5 wt.-% cellulose. Via NMR spectroscopy of the solution, we rule out chain degradation or derivatization, even after several weeks at elevated temperature. Based on our MD simulations, we estimate the enthalpy of solvation and derive a simple model for semi-quantitative prediction of cellulose solubility in ILs. With the help of Sankey diagrams, we illustrate the hydrogen bond network topology of the solutions, which is characterized by competing hydrogen bond donors and acceptors. The hydrogen bonds between cellulose and the anions possess average lifetimes in the nanosecond range, which is longer than found in common pure ILs.

## 1. Introduction

Within the last few decades of our century, renewable fuels, energies, and materials have gained increasing attention [1]. With an annual production of around $10^{11}$–$10^{12}$ tons, cellulose is by far the most abundant natural material on Earth [2]. It is obtained in significant amounts as a by-product in the agricultural and food industry, where it is mostly pyrolyzed [3]. However, cellulose possesses some very interesting physical and chemical properties that are advantageous for certain technological and industrial applications [4,5,6,7,8]. For example, cellulose ethers and esters [1,9] have been applied as additives in construction materials, paint, pharmaceutical products, and even in food [10,11,12].

**Figure 1:**
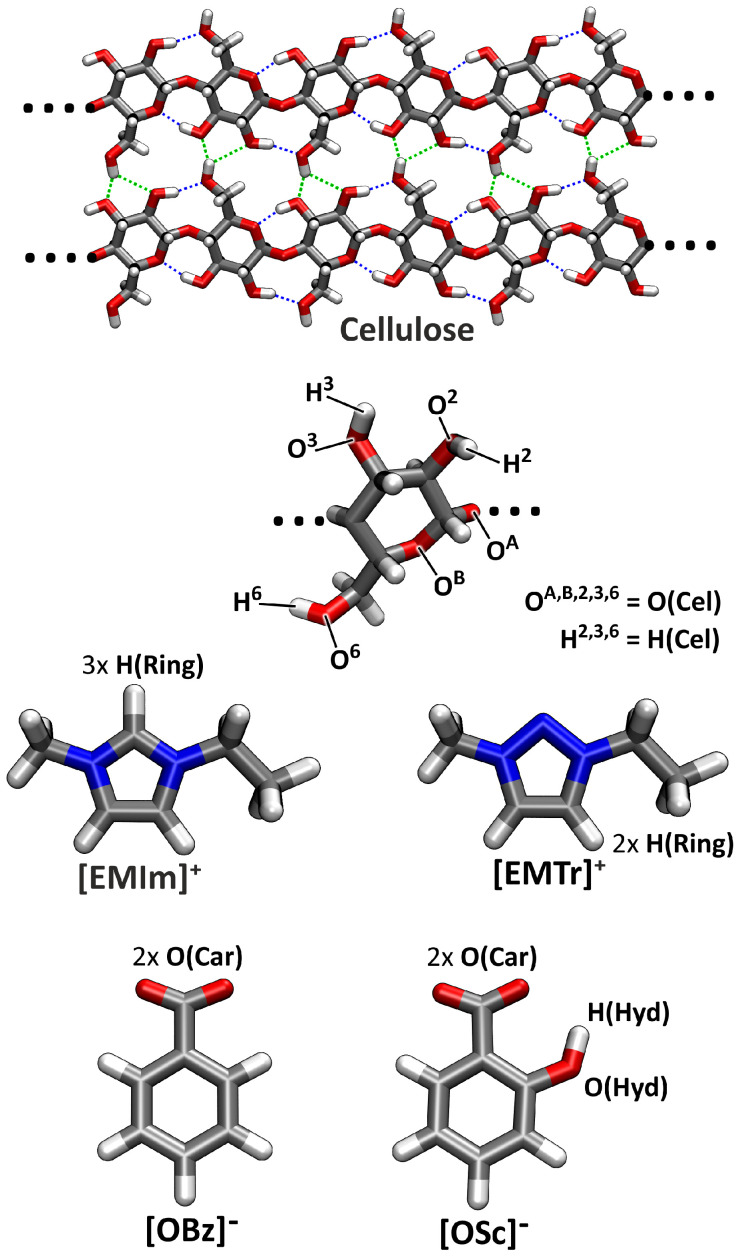
Molecular structures, nomenclature, and atom labels of the substances investigated in this study. The top panel shows a fragment from the crystal structure of cellulose I${}_{\beta }$ with intermolecular hydrogen bonds in green and intramolecular hydrogen bonds in blue. For abbreviations, see the text.

Cellulose is a polysaccharide with a degree of polymerization (DP) of around 200–10,000 units in its natural form, as produced by plants. It possesses a semi-crystalline structure and a significant amount of intramolecular hydrogen bonds, which contribute to the stiffness of the cellulose chains. These linear chains form two-dimensional sheet structures due to intermolecular hydrogen bonding [13] (see the top panel in Figure 1). The sheets, in turn, are held together by van der Waals interactions [14]. Due to the strong intermolecular hydrogen bond network and the high DP, it is a complicated task to dissolve cellulose, and most conventional solvents are not able to do so. However, processing techniques such as chemical modification often require bringing cellulose to solution first [5], which underlines the great practical importance of having efficient cellulose solvents [15].

In 2002, Swatloski et al. discovered that certain ionic liquids (ILs) are able to dissolve significant amounts of cellulose [10]. In principle known for more than 100 years now [16], ILs are a class of organic salts with a melting point below 100 °C. They often feature properties such as low vapor pressure, low toxicity, and good thermal stability [17,18,19] and possess a wide scope of applications in scientific and industrial fields [20,21], medicine [22,23], electrochemistry [24], as solvents [12], and in organic synthesis [18]. In order to dissolve cellulose, a solvent needs to break up the hydrogen bond network between the polymer strands [25] and replace it by hydrogen bonds to the solvent [8]. Most of the ILs proposed as cellulose solvents are based on imidazolium cations [10,25,26,27,28], which is not surprising, as it has been known for long that many imidazolium-based ILs form very strong hydrogen bonds [29,30,31,32,33,34,35,36]. Some of these imidazolium-based ILs are able to dissolve more than 20 wt.-% of cellulose if the anions are strong hydrogen bond acceptors [3,37,38].

However, there is also a drawback when using these ILs to dissolve cellulose. Anions with strong hydrogen bond accepting strength are very basic and can easily abstract a ring proton from the imidazolium cation, leading to the formation of *N*-heterocyclic carbenes (NHCs) [39]. These, in turn, are very reactive and lead to all kinds of unwanted by-products. This effect has indeed been utilized for some applications, such as the chemisorption of carbon dioxide [40] or even the dissolution of elemental sulfur [41] in ILs due to the reaction with intrinsically present NHCs. In the latter case, around 50 mol-% of the cations in the IL reacted with sulfur to form the carbene adducts within 24 h at 25 °C, emphasizing the importance of this reaction pathway. Such a reactivity is clearly unwanted in the case of dissolving cellulose; it has been reported that NHCs can lead to a molar mass reduction of cellulose [2], i.e., a degradation of the polymer.

Last year, we proposed triazolium-based ILs as a novel class of cellulose solvents [38]. This class of ILs has been discussed in the literature earlier [1,19,21,42,43], but had never been applied to dissolve cellulose before. The 1,2,3-triazolium cation is very similar to imidazolium; only a C–H fragment in the aromatic ring is replaced by a nitrogen atom. However, the former no longer possesses an isolated ring proton, leading to a significantly increased p$K_{a}$ value of around 24 when compared to imidazolium (p$K_{a}\approx 20$) [44]. As the p$K_{a}$ value of the cation determines the amount of NHC formation, triazolium-based ILs contain a significantly smaller amount of intrinsic carbenes.

After having introduced a new class of cations for ILs as cellulose solvents in our last article [38], we set the focus on the anions in this study. We combine the two previously studied cations 1-ethyl-3-methylimidazolium ([EMIm]${}^{+}$) and 1-ethyl-3-methyl-1,2,3-triazolium ([EMTr]${}^{+}$) with the two aromatic anions benzoate ([OBz]${}^{-}$) and salicylate ([OSc]${}^{-}$), leading to the four ionic liquids [EMIm][OBz], [EMIm][OSc], [EMTr][OBz], and [EMTr][OSc]. While the former two have already been characterized in the literature before [45,46,47], the latter two did not yet appear in the literature. All four ILs have never been applied to dissolve cellulose up to now. The four ions together with the nomenclature used here are visualized in the lower part of Figure 1.

**Figure 2:**
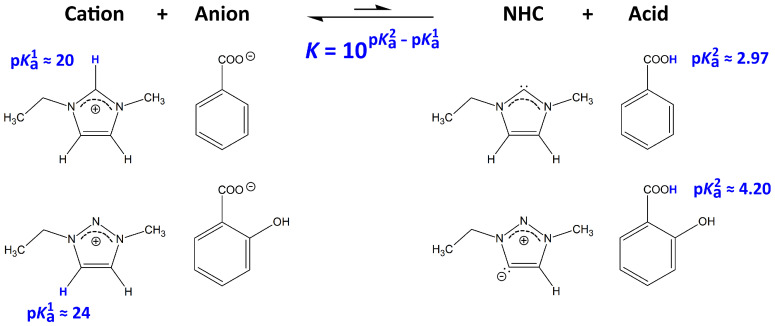
Process of *N*-heterocyclic carbene (NHC) formation for the ions involved in this study. The transferred protons are shown in blue color. p$K_{a}$ values are from the literature [44]. While p$K_{a}^{1}$ describes the acidity of the cation, p$K_{a}^{2}$ depicts the acidity of the acid corresponding to the anion after protonation. From the equation below the arrow, the equilibrium constant *K* of this process can be computed from the choice of anions and cations.

A scheme of the NHC formation in ILs is presented in Figure 2. While a direct spectroscopic determination of the intrinsically present amount of carbenes is unfortunately not possible with the methods available to us due to the small equilibrium concentration, we refer to the acid and base constants of the ions in our argument. While p$K_{a}^{1}$ describes the acidity of the cation, p$K_{a}^{2}$ depicts the acidity of the acid corresponding to the anion after protonation. As mentioned above, the [EMTr]${}^{+}$ cation lacks the isolated ring proton, which is present in imidazolium, and therefore possesses a significantly larger p$K_{a}$ value. The equation below the arrow in Figure 2 states that the equilibrium constant *K* of the carbene formation also depends on the basicity of the anion (the base constant of the anion and the acid constant of the corresponding acid are related by $pK_{a}+pK_{b}=pK_{W}=14$). The two anions investigated in this study, benzoate and salicylate, both possess a lower basicity than acetate, which is the anion in many ILs that have been used to dissolve cellulose before. Therefore, our choice of anions will even more reduce the amount of NHCs intrinsically present in equilibrium and therefore lead to even less unwanted by-products when dissolving cellulose.

The article is structured as follows. After a discussion of the physicochemical properties of the four ILs synthesized here, we investigate the cellulose solubility of the compounds and compare it to earlier studies. To better understand the results from these two parts, we complement our measurements by force field molecular dynamics simulations, which give us insight into the structure and dynamics of the hydrogen bond network and furthermore allow an estimate of the enthalpy of solvation. Subsequently, the experimental and computational methods are described. The article ends with conclusions.

## 2. Results and Discussion

### 2.1. Physicochemical Properties of the ILs

In this study, we synthesized four ILs based on the aromatic anions benzoate and salicylate. All four ILs were obtained as viscous liquids at room temperature. The purity of [EMIm][OBz], [EMIm][OSc], [EMTr][OBz], and [EMTr][OSc] was confirmed by ${}^{1}$H and ${}^{13}$C NMR spectroscopic measurements, as shown in the Supporting Information. In Table 1, we present the measured physicochemical properties of the ILs. For all substances, the water content was determined by Karl Fischer titration. In three cases, the water content was found to be below 1 %; only [EMTr][OSc] possesses a water content of around 1.3 %. The decomposition temperatures of the triazolium-based ILs are lower than those of the imidazolium-based ILs. [EMTr][OBz] and [EMTr][OSc] possess a decomposition temperature of 141 °C and 171 °C, respectively, while [EMIm][OBz] and [EMIm][OSc] decompose at slightly higher temperatures (see Table 1). The triazolium-based ILs show a higher viscosity when compared to the ILs with imidazolium cation. It should be noted that [EMTr][OSc] with a viscosity of 1.388 Pa s at *T* = 20 °C possesses the highest viscosity of the substances studied here, and [EMIm][OBz] has the lowest viscosity of 0.479 Pa s at *T* = 20 °C. Furthermore, Table 1 depicts the decrease of viscosity with increasing temperature (data for different temperatures are presented in the SI). At *T* = 90 °C, [EMTr][OBz] and [EMTr][OSc] possess a similar viscosity of around 0.025 Pa s. [EMTr][OSc] is found to have the highest density of the four compounds, with a value of 1.16 g cm${}^{-3}$ at room temperature. With increasing temperature, the densities decrease in all four cases. The physicochemical characterization indicates that the choice of the cation has a significant influence on the properties of the IL. The triazolium-based ILs possess both higher densities and higher viscosities when compared to their imidazolium-based counterparts.

### 2.2. Microstructure and Dynamics of the ILs

In order to understand the microscopic properties of the newly synthesized ILs, we performed force field molecular dynamics (MD) simulations of the liquids at 80 °C. For details on the simulations, see Section 3.2. It should be noted that the computations are not based on any results measured in this study; the force field parameters are either taken from the literature or obtained from ab initio calculations.

**Figure 3:**
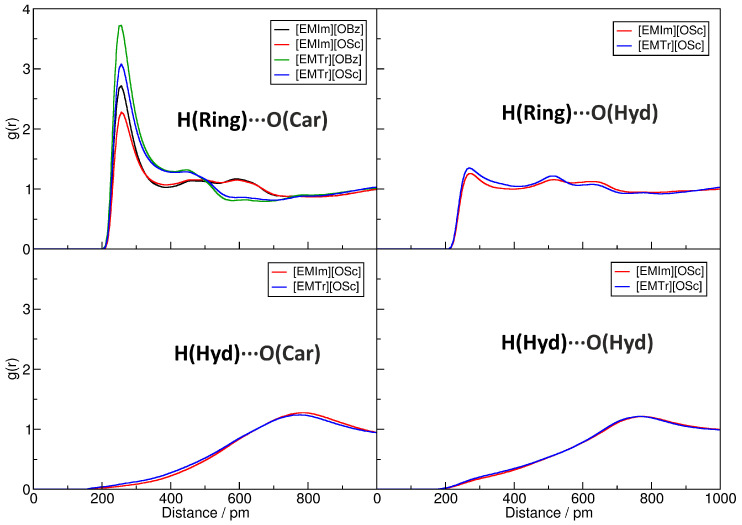
Computed radial distribution functions (RDFs) for the four pure ILs from the MD simulations. Peaks at 200…300 pmwith a height > 1 indicate hydrogen bonds. For the abbreviations, see Figure 1.

It is well known that hydrogen bonds play a significant role in imidazolium- and triazolium-based ILs [29,30,31,32,33,34,35,38]. Figure 3 presents the radial distribution functions (RDFs) between several hydrogen bond donors and acceptors in the investigated systems (for the atom labels, see Figure 1). The RDFs are normalized to uniform density: a value below one indicates a depletion of the observed atom type at the given distance, while values above one stand for an enrichment. Hydrogen bonds are characterized by a distinct peak at O⋯H distances of around 200 to 300 pm. In the upper left panel of Figure 3, the RDFs corresponding to the hydrogen bonds between the imidazolium and triazolium ring protons and the carboxylate oxygen atoms of the anions are depicted. It is clearly visible that all four ILs possess a distinct peak at typical hydrogen bond distances here, and we therefore can deduce that hydrogen bonding between these atoms is strong and important in the liquids. The intensity of the hydrogen bond is stronger for triazolium cations when compared to imidazolium and is slightly stronger for benzoate anions in comparison to salicylate. While the former observation can be understood from the fact that there are less ring protons in triazolium, and therefore each single interaction is more intense, the latter finding is due to the competition for the hydrogen bond acceptors because of intramolecular hydrogen bonding in salicylate. The competition between hydrogen bond donors and acceptors will be further discussed in Section 2.4. The position of the peak is around 230 pm for all systems, as is expected for aromatic C–H⋯O hydrogen bonds.

The upper-right panel of Figure 3 shows the RDFs for the hydrogen bond between the cations’ ring protons and the hydroxyl oxygen atom in the salicylate anion. There is a small peak visible at a distance of around 230 pm. The height of the peak is barely above one, which indicates a rather weak hydrogen bond between these atoms. The RDFs in the lower two panels of the Figure correspond to hydrogen bonds originating from the hydroxyl proton in salicylate as a donor. In both cases, there is no peak visible at typical hydrogen bond distances, and we can conclude that these types of hydrogen bonds are non-existent in our simulations. This is due to the fact that the hydroxyl proton forms an intramolecular hydrogen bond to one of the carboxylate oxygen atoms in salicylate, which is very strong and exists almost all the time [48,49]. Therefore, this proton is not available for intermolecular hydrogen bonds.

After having discussed the structure of the hydrogen bonds in the ILs, we will now investigate their dynamics. A reliable and well-established way to compute average hydrogen bond lifetimes is the autocorrelation approach [50,51] as implemented in the TRAVIS program package [52,53]. This approach is based on a geometric criterion to define if a hydrogen bond exists at a given time. We use a simple O⋯H distance criterion of 300 pm here. The resulting intermittent hydrogen bond lifetimes are given in Table 2. For the strong hydrogen bond between the cations’ ring protons and the carboxylate oxygen atoms in the anions (second column), we find lifetimes in the order of 1000 ps, which is rather long when compared to other results from simulation studies of ILs [31,32,35]. This indicates a permanent and only slowly varying hydrogen bond network between the anions and cations of the IL, which is in line with the experimentally observed high viscosities (see Table 1). We find the hydrogen bonds from triazolium to live longer than those originating from imidazolium, while the hydrogen bonds to benzoate live longer than those to salicylate. These observations are very well in line with the hydrogen bond intensities in the RDFs discussed above.

The hydrogen bond between the cations’ ring protons and the hydroxyl oxygen atom in salicylate, which was already found to be rather weak above, possesses lifetimes of around 200 ps (third column of the table), which is around a factor of five smaller than in the previous case. For completeness, we also computed the average lifetimes of the hydrogen bonds originating from the hydroxyl proton in salicylate, which were found to be almost non-existent above (last two columns of the table). They are all in a range of 20 to 50 ps, and therefore, as expected, significantly shorter lived than the previously discussed hydrogen bonds.

Another interesting property that is directly accessible from MD simulations is the self-diffusion coefficient of the system’s ingredients. These are computed from the slope of the mean squared displacement of the ions’ centers of mass. The results for the anions and cations in the four studied ILs are shown in Table 3. We find that the anions possess a slightly larger self-diffusion coefficient than the cations, as was observed in simulation studies of imidazolium-based ILs before [35]. Imidazolium cations diffuse faster than triazolium cations (in agreement with the longer hydrogen bond lifetimes for the latter ones), while benzoate anions diffuse faster than salicylate anions. It should be noted that the values are all rather low when compared to molecular liquids: water possesses a self-diffusion coefficient of around 6600 $\times 10^{-12}$ m${}^{2}$ s${}^{-1}$ at the same temperature [54]. This is in line with the experimentally observed high viscosities of the ILs (see above).

**Figure 4:**
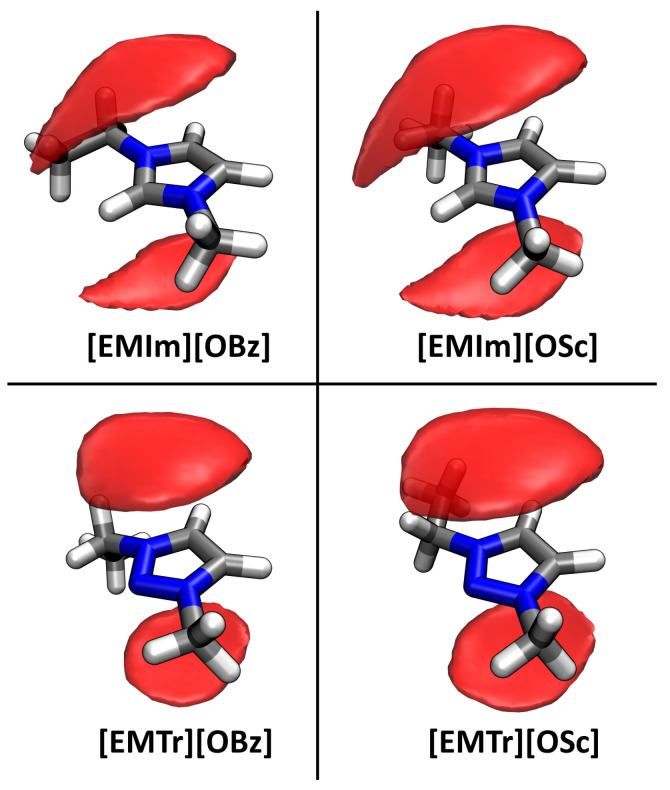
Spatial distribution function (SDF) of the anions’ ring centroids around the cation, shown at an isovalue of 12 nm${}^{-3}$. Aromatic ring stacking between anion and cation is clearly visible.

Finally, we turn our focus to π–π stacking of the aromatic ions. This effect has already been discussed much in the literature for liquids containing aromatic rings [55,56,57,58]. Despite the repulsive interaction due to the positive charge, imidazolium cations have been found to adopt configurations with π–π stacking in ILs. In our case, both the anions and cations are aromatic, so a very strong stacking of these rings can be expected (supported by the attractive Coulomb interaction). To investigate this claim, we computed spatial distribution functions (SDFs) with the TRAVIS program package [52,53]. In Figure 4, the probability of finding the ring center of an anion is presented as an isosurface in a local coordinate system fixed at the cation, averaged over all cations and the complete trajectory length. As can be seen from the plots, there is a very distinct localization of the anions’ ring centroids on top and below the cation’s aromatic ring. This confirms the presumption of very strong π–π stacking between the anions and cations in the four ILs. For the imidazolium-based cations, there is a slight offshoot of the distribution towards the isolated ring proton, while for the triazolium-based cations, the distribution of the anions’ ring centroids is symmetrically located above and below the triazolium ring. The distribution is slightly wider (and therefore less localized) for salicylate anions when compared to benzoate.

### 2.3. Cellulose Solubility

**Figure 5:**
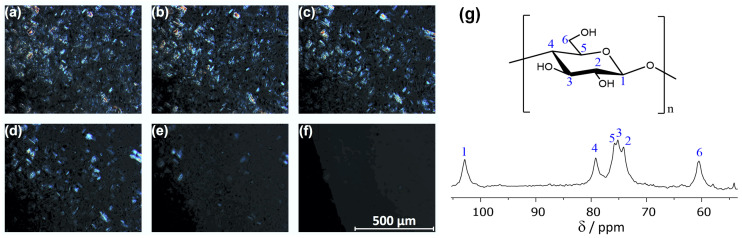
(**a**–**f**) Polarized optical microscopy (POM) images showing the dissolution of microcrystalline cellulose in [EMTr][OBz] at *T* = 60 °C (4 wt.-% cellulose in 200 mg [EMTr][OBz]). The pictures were taken directly after addition and 5, 10, 15, 30, and 135 min afterwards. (**g**) ${}^{13}$C NMR spectrum of 4 wt.-% cellulose in [EMTr][OBz]/DMSO–d${}_{6}$ at 45 °C taken at 125 MHz.

To visualize the dissolution of cellulose, we dissolved microcrystalline cellulose in [EMTr][OBz] at *T* = 60 °C and monitored the process by polarized optical microscopy (see Figure 5a–f). It is well visible how the cellulose crystals dissolve over time. The right-hand panel (g) of Figure 5 depicts the measured ${}^{13}$C NMR spectrum of the obtained solution after three weeks at *T* = 80 °C. The spectrum shows all characteristic NMR resonances of cellulose, indicating that the polymer strands remain intact in solution, and no significant amount of derivatization or decomposition occurs even after a relatively long time.

The measured cellulose solubility of the four ILs is presented in Table 4. For [EMTr][OBz] and [EMTr][OSc], solubility limits of 8.5 wt.-% and 4.8 wt.-% were obtained at *T* = 80 °C, respectively. In the case of [EMIm][OBz] and [EMIm][OSc], the spread is slightly larger, and the solubility limits were found to be 7.4 wt.-% and 2.9 wt.-% of cellulose at the same temperature, respectively. All measurements were performed with 200 mg of the corresponding IL.

Based on our force field MD simulations, we predicted the solvation enthalpy $\Delta H_{\mathrm{Solv}}$ of cellulose in the four ILs, normalized to one glucose unit of the cellulose strand; see Column 4 in Table 4. The solvation enthalpy is the energy that is released when one single strand of cellulose is transferred from vacuum into the IL, normalized to one glucose unit of the cellulose strand. It does not include the energy required to break up the cellulose crystal structure; however, this value is a constant for cellulose, and we are only interested in the differences in solvation enthalpy here. Details on the protocol used for the computation can be found in Section 3.2. It is already visible that there exists a common trend in experimental cellulose solubility and predicted solvation enthalpy. While [EMIm][OBz] possesses both the highest cellulose solubility and the largest solvation enthalpy, [EMIm][OSc] is found to have both the smallest cellulose solubility and the smallest solvation enthalpy, with the two triazolium-based ILs found between these two cases. In Columns 5 and 6 of Table 4, the contributions of the cellulose–anion and cellulose–cation interactions to the total potential energy are given. We find that the cellulose–anion interaction is significantly stronger than the cellulose–cation interaction, as has already been discussed before in the literature [38,59]. Therefore, the choice of the anion is of primary importance for the cellulose solubility of an IL. The details of the interactions between cellulose and the ILs will be discussed in Section 2.4. Note that the sum of the cellulose–anion and cellulose–cation interaction energies does not equal the solvation enthalpy, because the latter additionally contains the (positive) energetic contribution to create a void of suitable shape for the cellulose strand in the solvent, which involves the breaking of many hydrogen bonds in the IL.

**Figure 6:**
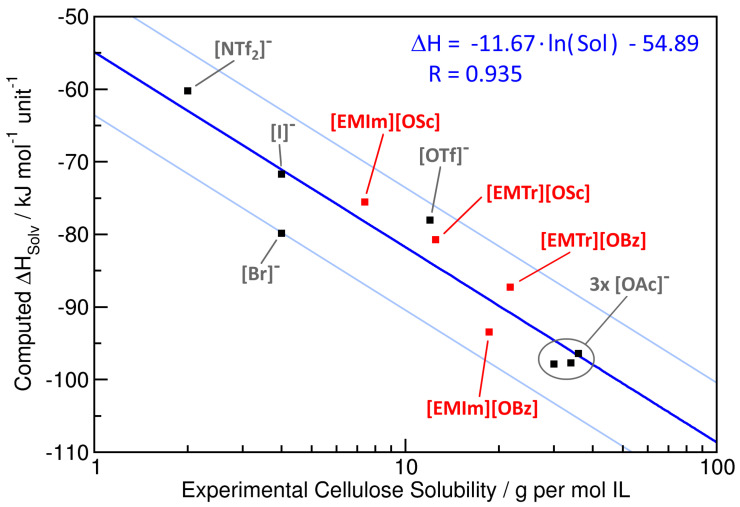
Correlation of the experimental cellulose solubility with the predicted enthalpy of solvation $\Delta H_{\mathrm{Solv}}$. The red data points correspond to the four ILs from this study, while the black points are taken from our recent article [38]. The blue curve is a regression function (equation in axis units and correlation coefficient R; see the upper right corner), and the two light blue curves depict a range of a factor of two deviation in predicted cellulose solubility.

To further investigate the correlation between experimental cellulose solubility and computationally predicted solvation enthalpy, we present a correlation plot for these two properties in Figure 6 (note the logarithmic horizontal axis for the cellulose solubility). While the four red points correspond to the ILs from this study, the seven black points have been taken from our previous article [38]. Already at first sight, a clear correlation between the quantities can be seen. A least-squares regression of the points is shown by the blue curve, and the corresponding equation is given in the upper-right corner of the plot (note that a logarithmic function appears as a straight line with a logarithmic horizontal axis). The correlation coefficient of the regression is R=0.935, indicating a rather good fit. The two light blue lines indicate the range of a factor of two deviation between predicted and experimental cellulose solubility. All points are located within this range. Therefore, we can conclude that our simple model can give a semi-quantitative prediction of cellulose solubility from molecular dynamics simulations, with a deviation between prediction and actual value less than a factor of two in all cases.

The reason for this correlation can be understood from the following consideration, which has already been briefly discussed in our previous article [38]. Dissolving cellulose in some solvent is an equilibrium process with equilibrium constant *K*. For such processes, it holds $\Delta G_{\mathrm{Solv}}=-R\cdot T\cdot \ln\left(K\right)$. Furthermore, we have $\Delta G_{\mathrm{Solv}}=\Delta H_{\mathrm{Solv}}-T\cdot \Delta S_{\mathrm{Solv}}$. For solvents with a similar molecular structure (as is the case for the ILs investigated here), it can be approximated that $T\cdot \Delta S_{\mathrm{Solv}}$ is constant for all the solvents, which we will call $c_{1}$ here. Therefore, we find the approximate relationship $\Delta H_{\mathrm{Solv}}-c_{1}\approx c_{2}\cdot \ln\left(K\right)$ with another constant $c_{2}$. This is exactly the relationship that is obtained from the regression above.

### 2.4. Microscopic Picture of Cellulose Solvation

To obtain a microscopic understanding of the interactions that cause the differences in cellulose solubility, we further analyze the trajectories obtained from our MD simulations of cellulose in the respective ILs in the following section. As already mentioned in the Introduction, a non-derivatizing cellulose solvent needs to break up the hydrogen bonds between the cellulose strands by forming strong hydrogen bonds to cellulose. In other words, dissolving cellulose is all about competition between different hydrogen bond donors and acceptors in the system. For a cellulose solution in [EMIm][OSc] or [EMTr][OSc], there are three different hydrogen bond donors—H(Ring), H(Hyd), and H(Cel)—and three different acceptors—O(Car), O(Hyd), and O(Cel)—present (see Figure 1 for atom labels). In the case of the two benzoate-based ILs, there are only two donors and acceptors, because H(Hyd) and O(Hyd) are missing. It is important to understand the topology of the hydrogen bond network in these systems, which is characterized by the competition between the donor and acceptor sites.

**Figure 7:**
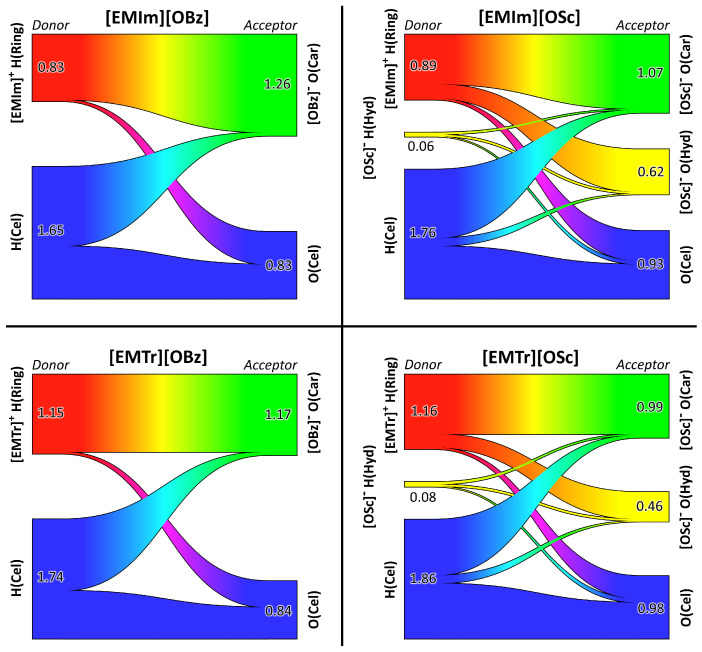
Sankey diagrams [53] visualizing the topology of the hydrogen bond network for the cellulose solution in the four ILs (one panel per IL). Hydrogen bond donors shown on the left side and acceptors on right-hand side. The numbers indicate the average hydrogen bond count per atom. For atom labels, see Figure 1.

To do so, we performed our recently developed hydrogen bond network topology analysis [53], which is based on Sankey diagrams [60]. The results are presented in Figure 7. Each panel corresponds to one of the four ILs investigated here. The hydrogen bond donors are placed on the left-hand side of each diagram and the acceptors on the right-hand site. The numbers depict the average count of hydrogen bonds formed by the donor/acceptor atom of the specified kind. The width of the bars is proportional to the corresponding numbers. Please note that the average numbers count the hydrogen bonds per atom, not per molecule. For example, an average number of 0.83 hydrogen bonds for the [EMIm]${}^{+}$ ring protons correspond to 0.83×3=2.49 hydrogen bonds on average donated by each [EMIm]${}^{+}$ ion. The different width of the bars on the left-hand and right-hand side in each diagram is a consequence of the different molecule count.

First, we will discuss the two diagrams on the left-hand side, corresponding to the two ILs with the benzoate anion. A significant amount of hydrogen bonding takes place between the cations’ ring protons and the anions’ carboxylate oxygen atoms, as already observed above for the pure ILs. In both [EMIm][OBz] and [EMTr][OBz], each carboxylate oxygen atom accepts around 1.2 hydrogen bonds on average. However, in the former liquid, each ring proton of the cation donates around 0.8 hydrogen bonds, while this number is around 1.2 in the latter system. This is a simple consequence of [EMIm]${}^{+}$ having one more ring proton, as $\frac{0.8}{1.2}\approx \frac{2}{3}$. Concerning the cellulose, slightly less than half of the donated and accepted hydrogen bonds are intramolecular (as the simulation contains only one cellulose strand, no intermolecular cellulose–cellulose hydrogen bonds can exist). These are the hydrogen bonds shown in blue color in the top panel of Figure 1. The other half of the hydrogen bonds formed by the cellulose donors and acceptors are formed to anions’ carboxylate oxygen atoms and cations’ ring protons, respectively. In the case of [EMTr][OBz], the cellulose hydrogen bond donors form significantly more hydrogen bonds in total, because more of the carboxylate oxygen atoms are free, as the cation only possesses two instead of three ring protons, which could occupy the oxygen atoms.

When considering the two ILs based on salicylate anions on the right-hand side of Figure 7, a similar picture emerges. Again, a significant amount of hydrogen bonding takes place between the cation’ ring protons and the anions’ carboxylate oxygen atoms. The hydroxyl proton of salicylate is donating only negligible amounts of intermolecular hydrogen bonds (0.06 and 0.08 on average), because it is occupied virtually all the time by a strong intramolecular hydrogen bond to one of the carboxylate oxygen atoms. This intramolecular hydrogen bond is deliberately excluded from the diagrams, because it would shadow the intermolecular hydrogen bonds of this proton. The hydroxyl oxygen atom in salicylate, on the other hand, accepts more hydrogen bonds on average, which are mostly donated by the cations’ ring protons. For the cellulose as well, a similar picture emerges. Around half of the hydrogen bonds donated and accepted by cellulose are intramolecular, while the other half is formed with anions’ carboxylate oxygen atoms and cations’ ring protons, respectively. Only a tiny amount of hydrogen bonding between cellulose and the hydroxyl group of salicylate is observed.

**Figure 8:**
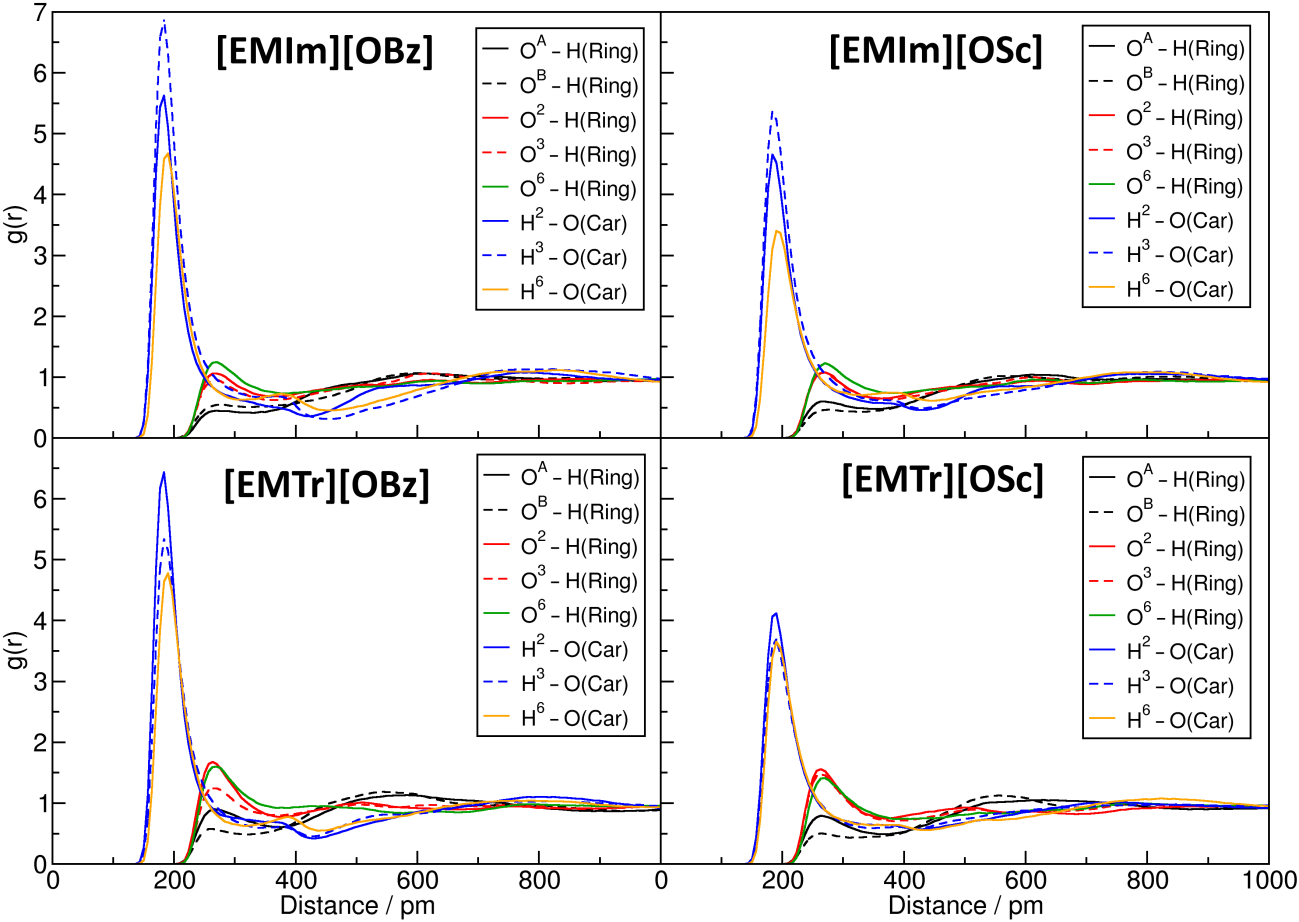
Radial distribution functions (RDFs) for the hydrogen bonds between cellulose and the anions and cations in the four systems. For atom labels, see Figure 1.

From this thorough investigation of the hydrogen bond network topology, we conclude that only the cations’ ring protons and the anions’ carboxylate oxygen atoms form relevant amounts of hydrogen bonds to cellulose, while the hydroxyl group of salicylate is negligible here. In a next step, we would like to find out if there are differences in the hydrogen bond strength for the different oxygen and hydrogen atoms in cellulose. To do so, we computed the relevant RDFs between the different sites in cellulose and the hydrogen bond donors and acceptors in the anions and cations. The results are presented in Figure 8. For the atom labels, please see Figure 1.

As a first observation, we note that the hydrogen bonds donated by cellulose and accepted by the anions’ carboxylate oxygen atoms—shown in blue and orange—are much shorter on average when compared to those donated by the cations’ ring protons and accepted by cellulose (peak position in the RDF of 200 pm compared to 260 pm). This is expected, because O–H⋯O hydrogen bonds are known to be significantly shorter than aromatic C–H⋯O hydrogen bonds, as the proton is much more polar in the former case. Furthermore, the height of the peak, and therefore the strength of the hydrogen bond, is much larger in the former case. All three hydrogen bond donor sites of cellulose behave similarly, with H${}^{6}$ exhibiting slightly weaker hydrogen bonds on average. The hydrogen bonds donated by cellulose are generally stronger in the benzoate-based ILs (left-hand side) when compared to the salicylate-based liquids (right-hand side), which is presumably due to the fact that the hydrogen bond acceptor abilities of salicylate are weakened due to the strong intramolecular hydrogen bond.

For the oxygen atoms of cellulose, larger differences are visible. The hydrogen bond acceptors O${}^{2}$, O${}^{3}$, and O${}^{6}$ form relatively strong hydrogen bonds to the cations’ ring protons, which are stronger for the triazolium-based ILs (lower two panels) when compared to the imidazolium-based liquids (upper two panels). The two acetal oxygen atoms O${}^{A}$ and O${}^{B}$, on the other hand, form significantly weaker hydrogen bonds to the cations’ ring protons, which is most probably due to the steric hindrance in the cellulose strand. Here also, the hydrogen bonds to [EMIm]${}^{+}$ are weaker than those to [EMTr]${}^{+}$.

After having discussed the structure of the individual hydrogen bonds of cellulose, the focus will now be on their dynamics and lifetimes. For the sake of clarity, we grouped together the donor and acceptor sites of cellulose that exhibit a similar behavior in Figure 8, as already indicated by identical coloring in that figure. To compute the average lifetimes of hydrogen bonds, the autocorrelation formalism was applied, as already described in Section 2.2. Again, a simple geometric distance criterion of 300 pm was used to define hydrogen bonds. The resulting intermittent lifetimes are given in Table 5.

In the table, several trends in the lifetimes can be observed. First, the hydrogen bonds donated by cellulose and accepted by the anions’ carboxylate oxygen atoms have significantly longer lifetimes when compared to those donated by the cations’ ring protons and accepted by cellulose. This was already expected due to the larger intensity and shorter average distances of the former group of hydrogen bonds in Figure 8. Among the cellulose hydroxyl protons, H${}^{2}$ and H${}^{3}$ form longer lived hydrogen bonds than H${}^{6}$. In the case of [EMIm][OBz], the average lifetime of the former hydrogen bond even exceeds 10 ns, which is an exceptionally long-living hydrogen bond (when, e.g., compared to those in common pure ILs [17,31,32,35,58]). It should be noted that such large lifetimes possess a very large statistical uncertainty, because the total production run of the simulation only amounted to 20 ns. The hydrogen bonds between cellulose and benzoate live longer on average than those between cellulose and salicylate, which is in line with the slightly lower peak height for the latter case in Figure 8. When considering the hydrogen bonds from the cations’ ring protons to the oxygen atoms of cellulose, the average lifetimes are significantly shorter, similar to those observed for the pure ILs above (see Table 2). Apart from small statistical deviations, the hydrogen bonds accepted by cellulose live longer in the triazolium-based ILs when compared to the imidazolium-based ILs, which is in line with the results in Figure 8. The lifetimes of the hydrogen bonds accepted by O${}^{2}$, O${}^{3}$, O${}^{A}$, and O${}^{B}$ are similar, while those accepted by O${}^{6}$ live slightly shorter. This is an interesting finding, as it is contrary to the structural results in Figure 8, where O${}^{6}$ was found to behave very similar to O${}^{2}$ and O${}^{3}$, while O${}^{A}$ and O${}^{B}$ were found to form significantly weaker hydrogen bonds. We consider this another example of the importance of investigating hydrogen bond structure and dynamics separately.

**Figure 9:**
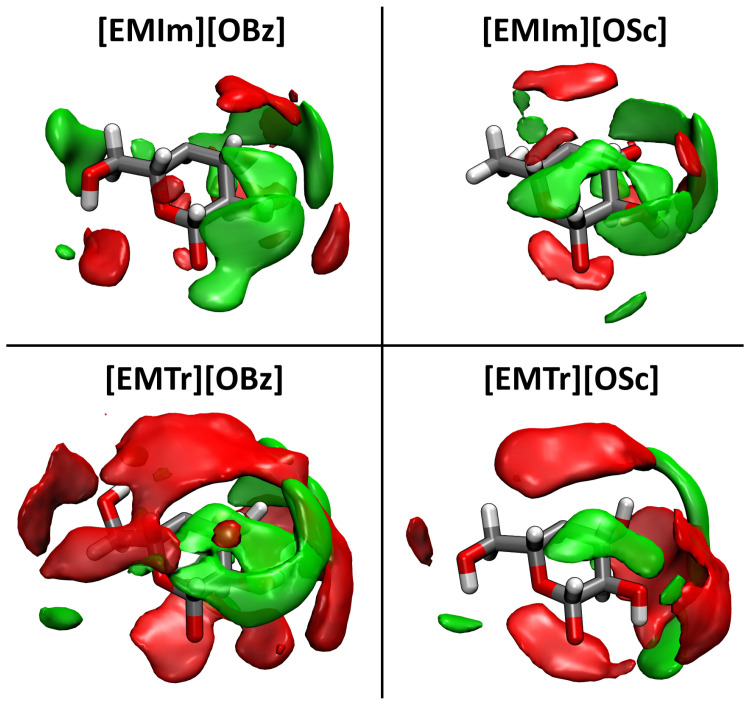
Spatial distribution functions (SDF) of H(Ring) (red, 7 nm${}^{-3}$) and O(Car) (green, 14 nm${}^{-3}$) around one monomer unit of the cellulose strand; averaged over all monomer units.

Finally, we want to take a closer look at the spatial distribution of the cations’ hydrogen bond donor sites and the anions’ hydrogen bond acceptor sites around cellulose. To do so, SDFs of these sites were computed in the local coordinate system of one glucose unit of the cellulose strand, averaged over all units in the strand. The results are presented in Figure 9, where each panel corresponds to one of the four ILs studied here. The red isosurfaces depict the distribution of the cations’ ring protons around cellulose, while the green isosurfaces indicate the distribution of the anions’ carboxylate oxygen atoms. It is visible that the localization of the ring protons is much more distinct in the case of the triazolium-based ILs, which is in line with the higher hydrogen bond intensity observed in these systems above. When comparing the benzoate-based and salicylate-based systems, as well as the distribution of the carboxylate oxygen atoms, there are no significant differences visible in the spatial distribution.

## 3. Materials and Methods

### 3.1. Experimental Details

#### 3.1.1. Materials

The compounds used for the synthesis, 1-ethyl-3-methylimidazolium chloride (>99%), methyl iodide (99.5%), potassium carbonate (≥99%), silver benzoate (99%), and sodium salicylate (≥99.5%), were received from Sigma Aldrich. Silver nitrate (≥99.5%) was purchased from Carl Roth. 1,2,3-Triazole (98%) and ethyl bromide (98%) were purchased from abcr GmbH. The solvents tetrahydrofuran (≥99.9%), dimethyl sulfoxide–d${}_{6}$ (99.8 atom-% D), acetone (≥99.5%), chloroform–d${}_{1}$ (99.8 atom-% D), and methanol (≥99%) were received from VWR, Armar Europa GmbH, Sigma Aldrich, and Carl Roth (water content below 100 ppm in all cases). Microcrystalline cellulose with a particle size of 20 μm and a molar mass of 37 kg mol${}^{-1}$ (as determined by viscosimetry) was purchased from Sigma Aldrich.

#### 3.1.2. Synthesis

The ionic liquids 1-ethyl-3-methylimidazolium benzoate ([EMIm][OBz]) and 1-ethyl-3-methylimidazolium salicylate ([EMIm][OSc]) were synthesized from 1-ethyl-3-methylimidazolium chloride ([EMIm][Cl]) [23,61]. At first, [EMIm][Cl] (20.46 mmol) was dissolved in 10 mL methanol and added to a solution of silver benzoate (20.46 mmol) in 10 mL methanol. The solution was stirred for 5 min under exclusion of light at room temperature. After the reaction had finished, the mixture was filtered, the solvent was removed, and the residue was dried under vacuum (400 μbar). [EMIm][OBz] was obtained as a viscous colorless liquid. Analogous to the synthesis route of [EMIm][OBz], we synthesized [EMIm][OSc]. [EMIm][Cl] (20.46 mmol) was dissolved in acetone (10 mL) and added to a solution of silver salicylate (20.46 mmol) in acetone (10 mL). The reaction mixture was stirred for 5 min under exclusion of light at room temperature. After filtration, solvent removal, and drying under vacuum, [EMIm][OSc] was obtained as a colorless viscous liquid.

1-ethyl-3-methyltriazolium benzoate [EMTr][OBz] and 1-ethyl-3-methyltriazolium salicylate [EMTr][OSc] were prepared from 1-ethyl-3-methyl-1,2,3-triazolium bromide ([EMTr][Br]). The synthesis involved an alkylation with methyl iodide [24] and ethyl bromide [19]. 1,2,3Triazole (0.22 mol) was dissolved in a suspension of potassium carbonate (0.43 mol) in 150 mL THF. To this solution, methyl iodide (0.43 mol) was added, and the mixture was stirred for 24 h at room temperature. After the reaction finished, the mixture was filtrated, the solvent was removed, and the residue was dried under vacuum (400 μbar). The 1-methyl-1,2,3-triazole, which was obtained as a yellow liquid, was dissolved in CHCl${}_{3}$ (20 mL) and washed with water (3 × 20 mL). After this process, the solvent was removed and the liquid was dried under vacuum. In the next step [38], 1-methyl-1,2,3-triazole (60.2 mmol) was dissolved in ethyl bromide (60.2 mmol) and stirred for 48 h at 85 °C. The product was dried under vacuum (400 μbar) and recrystallized from toluene. [EMTr][Br] (10.4 mmol) was dissolved in methanol (10 mL) and added to a solution of silver benzoate (10.4 mmol) or silver salicylate (10.4 mmol) in methanol (10 mL). The reaction mixture was stirred for 10 min under exclusion of light at room temperature. After the reaction had finished, the solution was filtered and the products were dried under vacuum (400 μbar). The ILs [EMTr][OBz] and [EMTr][OSc] were obtained as viscous liquids. The characterization of [EMIm][OBz], [EMIm][OSc], [EMTr][OBz], and [EMTr][OSc] via ${}^{1}$H and ${}^{13}$C NMR spectroscopy can be found in the Supporting Information.

The silver salicylate used in the above syntheses was prepared from sodium salicylate [62]. A solution of sodium salicylate (62.5 mmol) in water (50 mL) was added drop-wise to 250 mL of an aqueous solution of silver nitrate (73.5 mmol). The colorless precipitate was collected, washed with water (3× 20 mL), and dried in vacuum (400 μbar).

#### 3.1.3. Equipment

The cellulose solubility experiments were performed under an atmosphere of dry nitrogen (water content < 0.5 ppm). To 200 mg of the respective IL at a temperature of 80 °C, 2 mg of microcrystalline cellulose were added and stirred for 24 h. When the cellulose dissolved completely within 24 h (as analyzed by polarized optical microscopy), another 2 mg of cellulose were added. The solubility limit was attained when finally the next 2 mg of cellulose could not longer be dissolved within 24 h. Polarized optical microscopy (POM) was carried out using an Axioplan 2 imaging microscope from Carl ZEISS Jena. The optical images were recorded with an Axio Cam MRc camera. The sample was placed between two cover slips separated by a Teflon spacer. The images were taken upon dissolution of cellulose at 60 °C in intervals of 5 min. The water content of the ILs was determined by Karl Fischer titration with a Mettler Toledo DL 35 titrator. For the titration, Hydranal Titrant 5 and Hydranal solvent oil from Honeywell/Fluka were utilized. The viscosity measurements were carried out in a temperature range of 20 °C ≤T≤ 90 °C and a shear rate of $10^{-1}$ Hz $\leq \dot{\gamma }\leq$ $10^{2}$ Hz, with an Anton Paar Physica MCR 301 shear rheometer. It was equipped with a CP25-2/TG measurement system having a cone-plate geometry with a diameter of 25 mm, an angle of 2°, and a gap of 51 μm. Using a DMA 60 density meter equipped with a DMA 602 measurement loop from Anton Paar, the liquid mass densities were measured. The measurement temperature was controlled by a thermostat from Julabo. The ${}^{13}$C NMR spectrum of cellulose in [EMTr][OBz]/DMSO–d${}_{6}$ (4 wt.-% of cellulose in IL, [EMIm][OBz]:DMSO–d${}_{6}$ 1:2 *v*/*v*) was recorded at 45 °C with an Agilent Technologies 500 MHz DD2 spectrometer operating at 125 MHz for ${}^{13}$C nuclei. 10,000 scans were recorded for a good signal-to-noise ratio, and the FID was apodized with an exponential function. Thermogravimetric measurements were performed with a heating rate of 10 K min${}^{-1}$ under continuous nitrogen flow. The mass loss was detected with a Mettler Toledo TGA/SDTA 851e module in the temperature range of 25 °C ≤T≤ 800 °C, and alumina pans were filled with about 5–15 mg of sample. The decomposition temperatures given in Table 1 are related to a weight loss of 5 wt-%.

### 3.2. Computational Details

The force field MD simulations were performed with the LAMMPS program package [63]. The initial configurations were prepared by Packmol [64]. For the imidazolium cations, the Canongia Lopes & Pádua (CL&P) force field was applied [65], which is a refinement of the OPLS–AAforce field [66] for ionic liquids. For the 1,2,3-triazolium cations, the force field parameters derived in our previous study [38] were applied. For benzoate and salicylate, all bonded interactions and Lennard-Jones parameters were taken from OPLS–AA [66], while the atomic charges (shown in Figure S2 in the SI) were derived from RESP calculations [67]. For cellulose, the force field parameters from Damm were used [68]. As in our previous study [38], all charges in the systems were scaled down by a factor of 0.8 to account for screening effects [69]. The integration time step was 0.5 fs in all cases. No bonds or angles were constrained. Lennard-Jones interaction as well as short-range Coulomb interactions were computed up to a cutoff distance of 800 pm. Long-range Coulomb interactions were computed via the PPPM approach as implemented in LAMMPS.

The simulation protocol was as follows. Starting from the initial configurations, an energy minimization was performed to ensure that no atoms were too close. The velocities were initialized to 500 K according to the Maxwell–Boltzmann distribution. After a short 25 ps run in the NVTensemble with temperature rescaling, the temperature was ramped down to 353 K during a subsequent 100 ps run, in which the temperature was controlled by a Nosé–Hoover chain thermostat [70,71,72] with a coupling constant of 100 ps. Then, the ensemble was switched to NpT. The pressure was controlled by a Nosé–Hoover barostat with a coupling constant of 2 ns. After a short NpT run of 100 ps for a first density equilibration, a Langevin dynamics run for 2 ns followed to dampen acoustic shock waves resulting from the change in cell size. Subsequently, a long NpT run of 5 ns was performed, and the system density was averaged over the run. Finally, the ensemble was switched back to NVT, and the simulation cell was re-sized to match the average density from the previous NpT run. After another Langevin dynamics run for 1 ns to dampen shock waves, a last equilibration for 1 ns followed, after which the production run of 20 ns took place.

The solvation enthalpies were estimated as follows. For each of the ILs, a simulation of 192 ion pairs of the pure IL, as well as a simulation of a cellulose strand with 5 glucose units in 192 ion pairs of the IL were performed, and the potential energy during the production run was recorded and averaged. 1000 snapshots of the cellulose strand from the previous simulations were taken, and from each of these snapshots, a simulation of the cellulose strand in vacuum was started (to ensure ergodicity). Based on these three averaged potential energies, the solvation energy was estimated as $\Delta H_{\mathrm{Solv}}\approx E_{\mathrm{Pot}}\left(\mathrm{Solution}\right)-E_{\mathrm{Pot}}\left(\mathrm{Pure}\;\mathrm{IL}\right)-E_{\mathrm{Pot}}\left(\mathrm{Cellulose}\;\mathrm{in}\;\mathrm{Vacuum}\right)$.

All trajectory analyses were performed by the TRAVIS program package [52,53]. The plots in Figure 3, Figure 6, and Figure 8 were created with Xmgrace [73], while Figure 1, Figure 4, and Figure 9 were rendered with VMD [74] and Tachyon [75]. Figure 7 was directly produced by TRAVIS [52,53].

## 4. Conclusions

In this combined experimental and computational study, we investigated imidazolium- and triazolium-based ILs with aromatic anions as cellulose solvents. We synthesized four ILs, which were the combinations of the 1-ethyl-3-methylimidazolium ([EMIm]${}^{+}$) and 1-ethyl-3-methyl-1,2,3-triazolium ([EMTr]${}^{+}$) cations with the benzoate ([OBz]${}^{-}$) and salicylate ([OSc]${}^{-}$) anions, i.e., [EMIm][OBz], [EMIm][OSc], [EMTr][OBz], and [EMTr][OSc]. While the former two ILs have already been characterized in the literature before [45,46,47], the latter two did not yet appear in the literature. Due to the lower basicity of the two anions when compared to acetate, the amount of intrinsically present *N*-heterocyclic carbenes (NHCs) in the ILs should be lower, which should lead to a lower reactivity and to a reduced amount of unwanted by-products. We characterized the ILs in terms of physicochemical properties such as density, viscosity, and decomposition temperature. All four ILs are liquids at room temperature. Based on force field MD simulations, we investigated the microstructure of the liquids and the hydrogen bond network. As expected for this class of substances, we found that hydrogen bonds play an important role within the intermolecular interactions. The hydrogen bond network is very persistent with average hydrogen bond lifetimes of up to 1 ns, which is in line with the high viscosity of the ILs. We observed a very strong π–π stacking interaction in the liquids. Such an interaction has been characterized before for ILs with aromatic cations, but is much stronger here because both the anion and cation are aromatic.

Next, we turned our attention to the ability of the ILs to dissolve cellulose. According to the literature, none of the four ILs has been applied to dissolve cellulose up to now. We measured their ability to dissolve cellulose and found that they dissolve between 2.9 and 8.5 wt.-% cellulose at 80 °C, which are relatively good values. By ${}^{13}$C NMR spectroscopy of the solutions after three weeks at 80 °C, we confirmed that the cellulose strands remain intact and no degradation or derivatization takes place even after relatively long times at elevated temperature. Based on our MD simulations, we estimated the solvation enthalpy $\Delta H_{\mathrm{Solv}}$ of cellulose in the ILs. We presented a simple regression model, which is able to semi-quantitatively predict the cellulose solubility based on the $\Delta H_{\mathrm{Solv}}$ values from the MD simulations. This protocol makes it possible to predict the ability to dissolve cellulose for new ILs before they even have been synthesized, so that a computational screening might be performed beforehand.

Finally, we analyzed our simulation trajectories to obtain a microscopic picture of the structure and dynamics of the solvation shell around cellulose. With our newly developed analysis of the hydrogen bond network topology based on Sankey diagrams, we gave a direct visualization of the competition between the different hydrogen bond donor and acceptor sites in the systems. We found that cellulose only forms hydrogen bonds to the ring protons of the cations and the carboxylate oxygen atoms of the anions, while the hydroxyl group of salicylate does not play a direct role here. The average lifetimes of the hydrogen bonds between cellulose and carboxylate oxygen atoms are particularly long-lived, with lifetimes of more than 10 ns in some cases, which is longer than found in common pure ILs.

## Figures and Tables

**Table 1:** Experimentally determined viscosity, density, water content, and decomposition temperature (5% weight loss) of the ionic liquids.

Ionic Liquid	Viscosity/Pa s		Density/g cm${}^{-3}$		State at RT	Water Content/%	Decomp.Temp./°C
	20 °C	90 °C	25 °C	85 °C			
[EMIm][OBz]	0.479	0.014	1.16	1.10	*liquid*	1.27	214
[EMIm][OSc]	0.495	0.017	1.20	1.15	*liquid*	0.65	234
[EMTr][OBz]	0.746	0.025	1.20	1.14	*liquid*	0.87	141
[EMTr][OSc]	1.388	0.024	1.25	1.19	*liquid*	0.33	171

**Table 2:** Computed intermittent hydrogen bond lifetimes from the MD simulations at 80 °C. An O⋯H distance criterion of 300 pm was used to define hydrogen bonds. For the atom labels, see Figure 1.

Ionic Liquid	Hydrogen Bond Lifetime/ps			
	H(Ring)⋯O(Car)	H(Ring)⋯O(Hyd)	H(Hyd)⋯O(Car)	H(Hyd)⋯O(Hyd)
[EMIm][OBz]	978	—	—	—
[EMIm][OSc]	664	208	22	18
[EMTr][OBz]	1140	—	—	—
[EMTr][OSc]	797	228	50	29

**Table 3:** Computed self-diffusion coefficients of the ions in the pure ILs from the MD simulations at 80 °C.

Ionic Liquid	Self-Diffusion Coefficient/$10^{-12}$ m${}^{2}$ s${}^{-1}$	
	Anion	Cation
[EMIm][OBz]	97.4	61.5
[EMIm][OSc]	108.9	69.6
[EMTr][OBz]	66.6	50.0
[EMTr][OSc]	74.6	52.7

**Table 4:** Experimentally determined cellulose solubilities at 80 °C (Columns 2 and 3); computed $\Delta H_{\mathrm{Solv}}$ per glucose unit (Column 4); contributions of the cellulose–anion and cellulose–cation interaction energies (Columns 5 and 6).

Ionic Liquid	Experimental Cellulose Solubility (80 °C)		Computed $\Delta H_{\mathrm{Solv}}$/kJ mol${}^{-1}$ unit${}^{-1}$	Interaction Energy/kJ mol${}^{-1}$ unit${}^{-1}$	
	g per mol IL	wt.-%		Cellulose–Anion	Cellulose–Cation
[EMIm][OBz]	18.6	7.4	−93.4	−114.8	−64.9
[EMIm][OSc]	7.4	2.9	−75.5	−105.1	−68.7
[EMTr][OBz]	21.7	8.5	−87.3	−120.7	−66.5
[EMTr][OSc]	12.5	4.8	−80.7	−100.0	−70.9

**Table 5:** Computed intermittent hydrogen bond lifetimes between cellulose and solvent. For atom labels, see Figure 1.

Ionic Liquid	Hydrogen Bond Lifetime/ps				
	O${}^{A},B\cdots$H(Ring)	O${}^{2},3\cdots$H(Ring)	O${}^{6}\cdots$H(Ring)	H${}^{2},3\cdots$O(Car)	H${}^{6}\cdots$O(Car)
[EMIm][OBz]	1059	1186	1182	10,730	2922
[EMIm][OSc]	709	842	516	3169	1288
[EMTr][OBz]	1320	1474	976	5869	4085
[EMTr][OSc]	2521	1783	579	3088	3560

## Supplementary Materials

The supplementary materials are available online.

## Abbreviations

The following abbreviations are used in this manuscript:

[EMIm]${}^{+}$	1-Ethyl-3-methylimidazolium cation
[EMTr]${}^{+}$	1-Ethyl-3-methyl-1,2,3-triazolium cation
[OBz]${}^{-}$	Benzoate anion
[OSc]${}^{-}$	Salicylate anion
CL&P	Canongia Lopes & Pádua force field
DP	Degree of polymerization
FID	Free induction decay
IL	Ionic liquid
MD	Molecular dynamics
MSD	Mean squared displacement
NMR	Nuclear magnetic resonance
NHC	*N*-heterocyclic carbenes
POM	Polarized optical microscopy
PPPM	Particle–particle particle–mesh method
RDF	Radial distribution function
RESP	Restrained electrostatic potential
SDF	Spatial distribution function
