# Supplementary material for: Dissolving Cellulose in 1,2,3-Triazolium- and Imidazolium-Based Ionic Liquids with Aromatic Anions
Source: Molecules. 2020 Aug 2;25(15):3539. doi: 10.3390/molecules25153539 (PMC7435399; doi:10.3390/molecules25153539)
Supplement: Supplementary file 1 [file molecules-25-03539-s001.pdf]

Supporting Information for

Dissolving Cellulose in 1,2,3-Triazolium- and Imidazolium-Based Ionic Liquids with Aromatic Anions

Martin Brehm[1*], Julian Radicke[1], Martin Pulst[1],
Farzaneh Shaabani[1], Daniel Sebastiani[1] & Jörg Kressler[1]

[1]Institut für Chemie, Martin-Luther-Universität Halle–Wittenberg,
von-Danckelmann-Platz 4, D-06120 Halle (Saale), Germany.

Materials and Methods

Synthesis of 1-ethyl-3-methyl-1,2,3-triazolium bromide:

The cation 1-ethyl-3-methyl-1,2,3-triazole [EMTr] was prepared from 1,2,3-triazole. At first, 1,2,3-triazole (0.43 mol) were dissolved in a suspension of potassium carbonate (0.86 mol) in tetrahydrofurane (750 mL). To this solution, methyl iodide (0.86 mol) was added and stirred at room temperature for 24 h. After the reaction, the mixture was filtrated and dried under vacuum. In the next step, ethyl bromide (60.2 mmol) was added to 1-methyl-1,2,3-triazole (60.2 mmol) and stirred for 48 h at 85 °C. After the reaction was finished, the product was dried under vacuum.

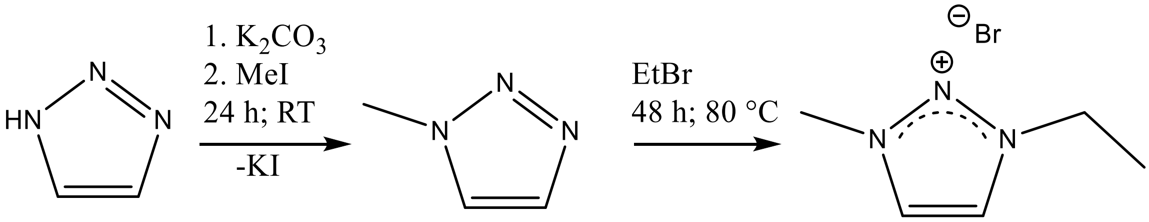

1-Methyl-1,2,3-triazole [MTr]

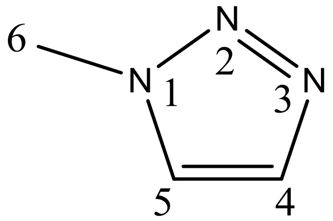

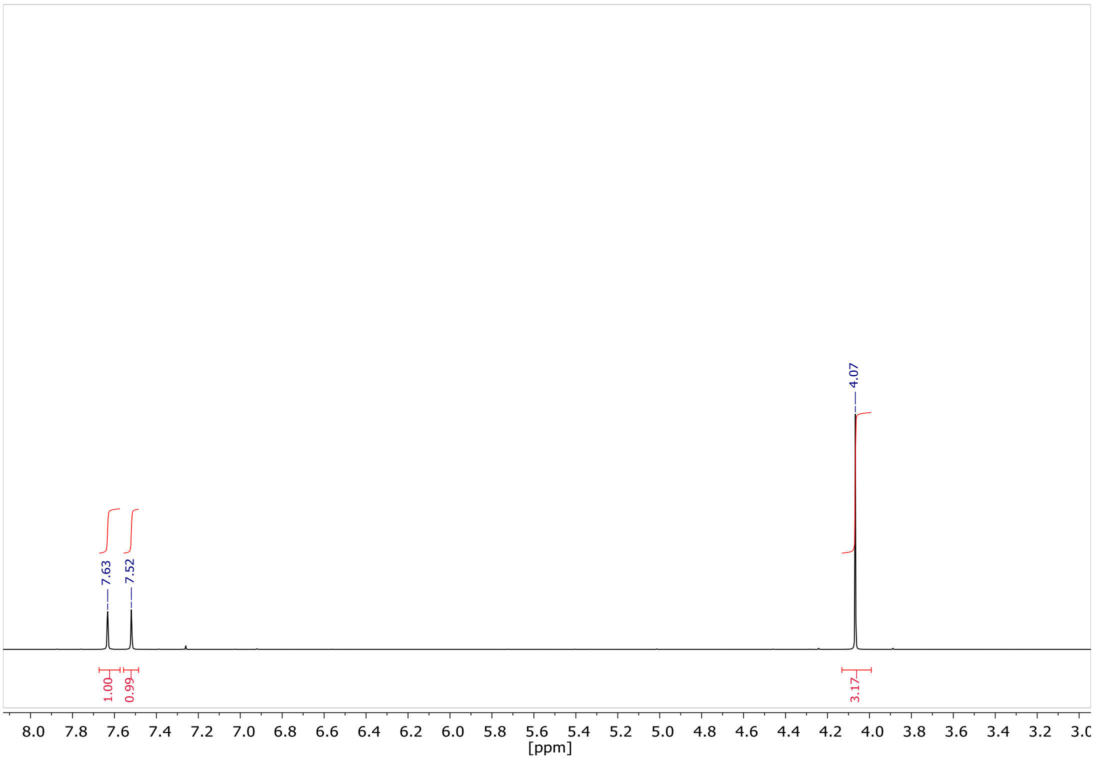

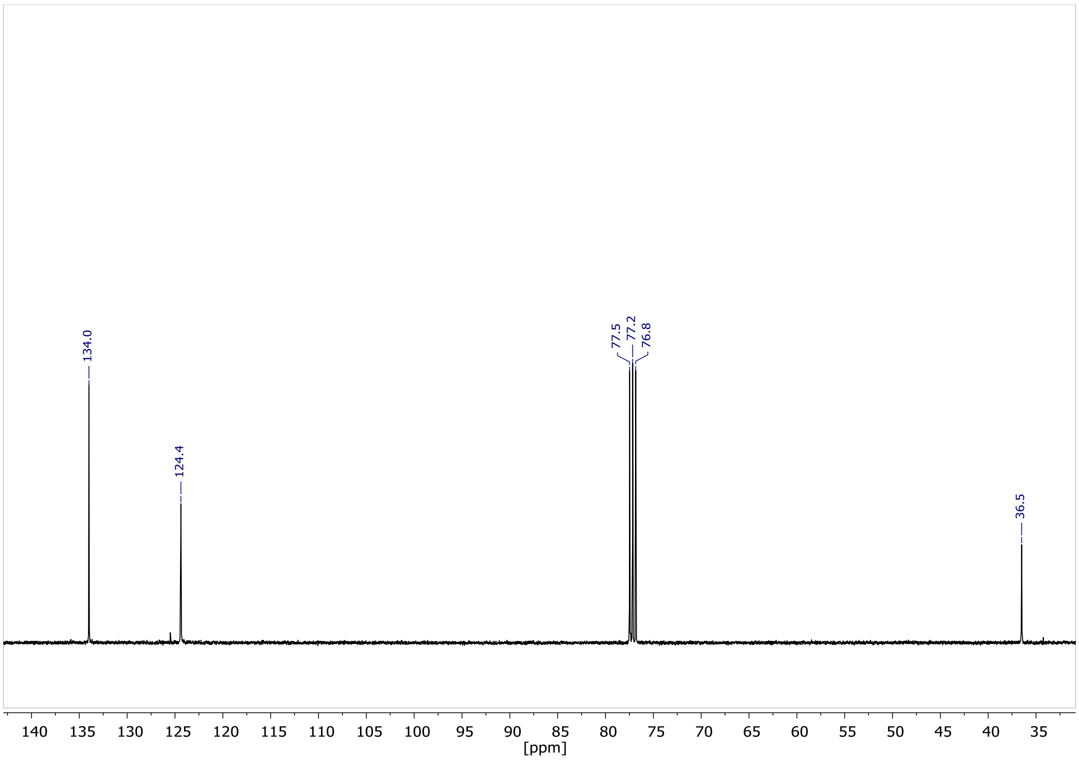

^{1}H NMR (500 MHz, $CDCl_3$, 27 °C): δ = 7.63 (*s*, 1H, H–4); 7.52 (*s*, 1H, H–5); 4.07 (*s*, 3H, H–6) ppm;

^{13}C NMR (126 MHz, $CDCl_3$, 27 °C): δ = 134.0 (C–4); 124.4 (C–5); 36.5 (C–6) ppm.

1-Ethyl-3-methyl-1,2,3-triazolium bromide [EMTr][Br]

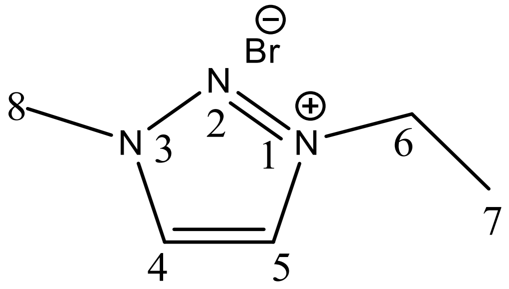

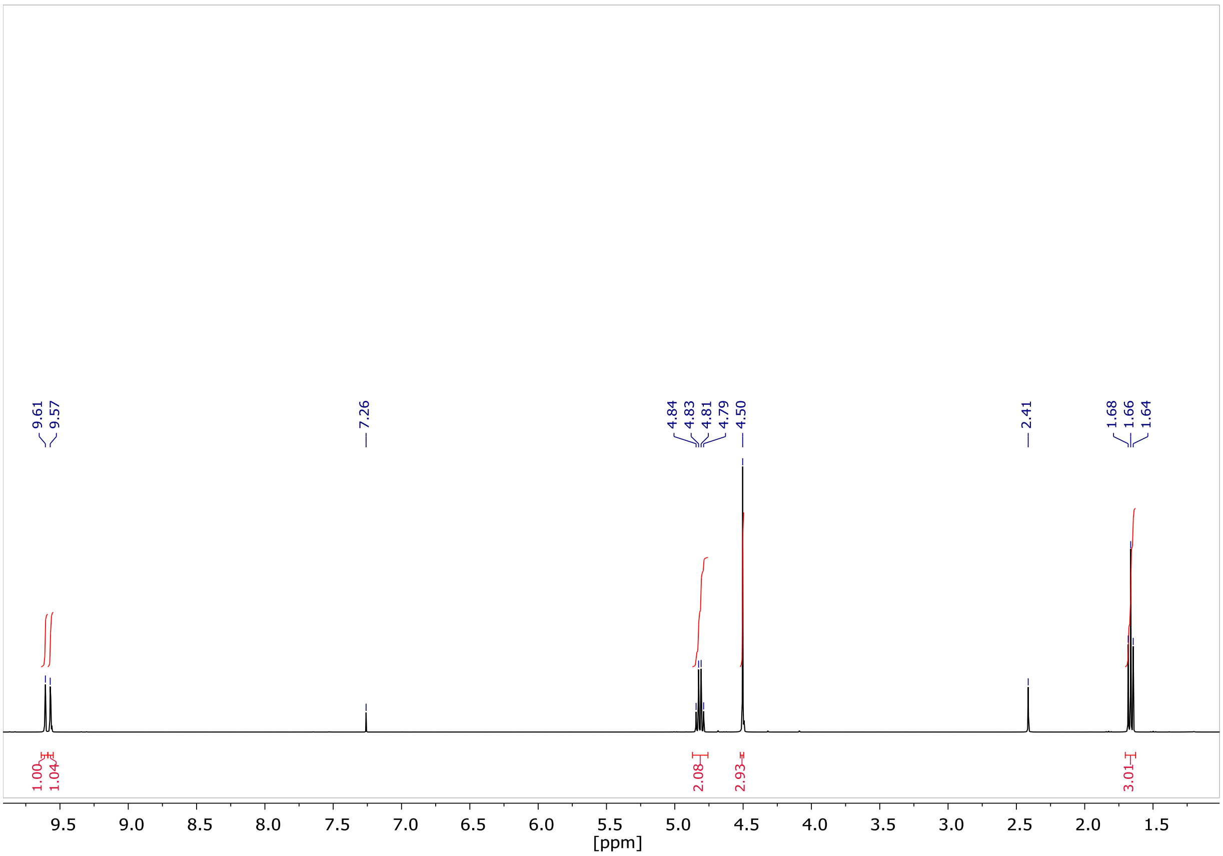

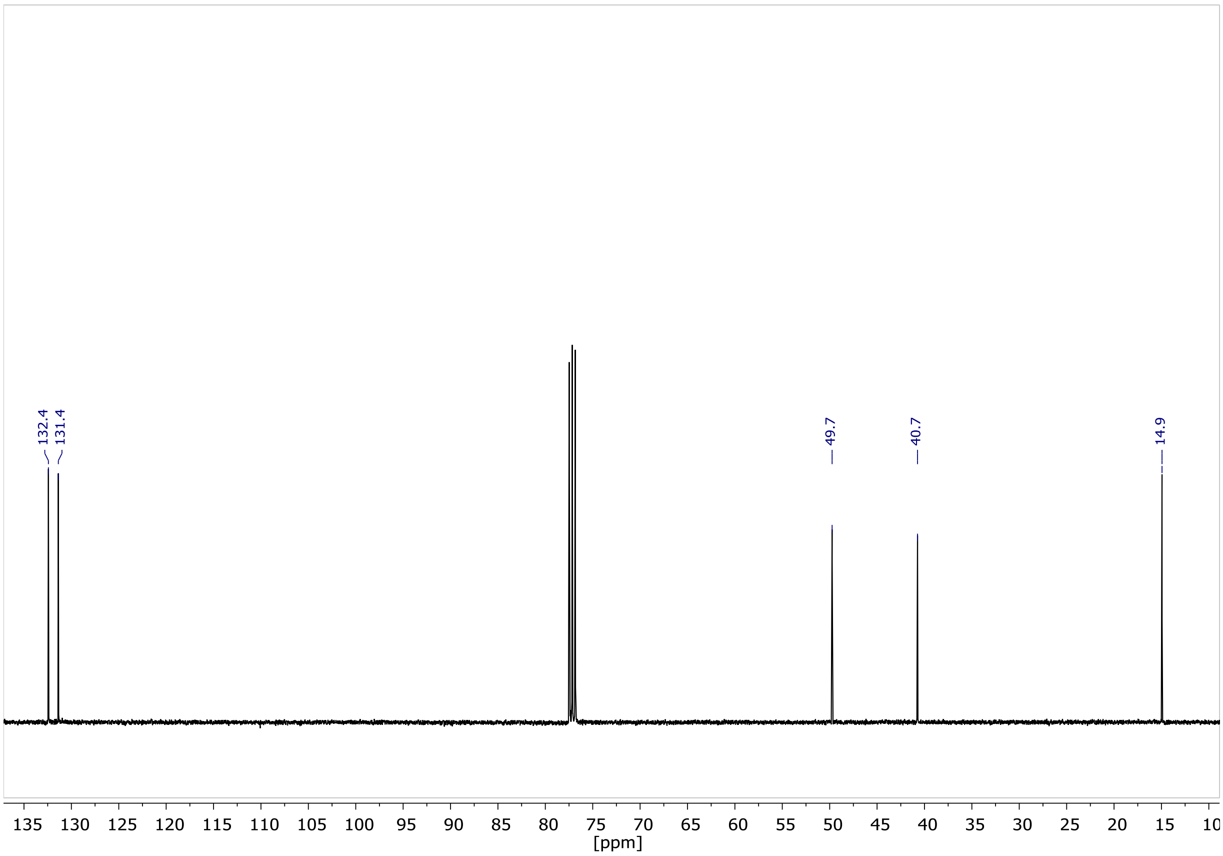

^{1}H NMR (500 MHz, $CDCl_3$, 27 °C): δ = 9.61 (*s*, 1H, H–4); 9.57 (*s*, 1H, H–5); 4.82 (*q*, 2H, ^{3}J = 7.4 Hz; H–6); 4.50 (*s*, 3H, H–8); 1.66 (*t*, 3H, ^{3}J = 7.4 Hz, H–7) ppm;

^{13}C NMR (126 MHz, $CDCl_3$, 27 °C): δ = 132.4 (C–4); 131.4 (C–5); 49.7 (C–6); 40.7 (C–8); 14.9 (C–7) ppm.

Synthesis of the triazolium-based ionic liquids:

1-ethyl-3-methyl-1,2,3-triazolium bromide (15.6 mmol) was dissolved in methanol (10 mL). To this solution, a solution of the silver derivate (15.6 mmol) in methanol (10 mL) was added dropwise. The reaction was stirred at room temperature for 10 min under exclusion of light. After this, the mixture was filtrated and dried under vacuum.

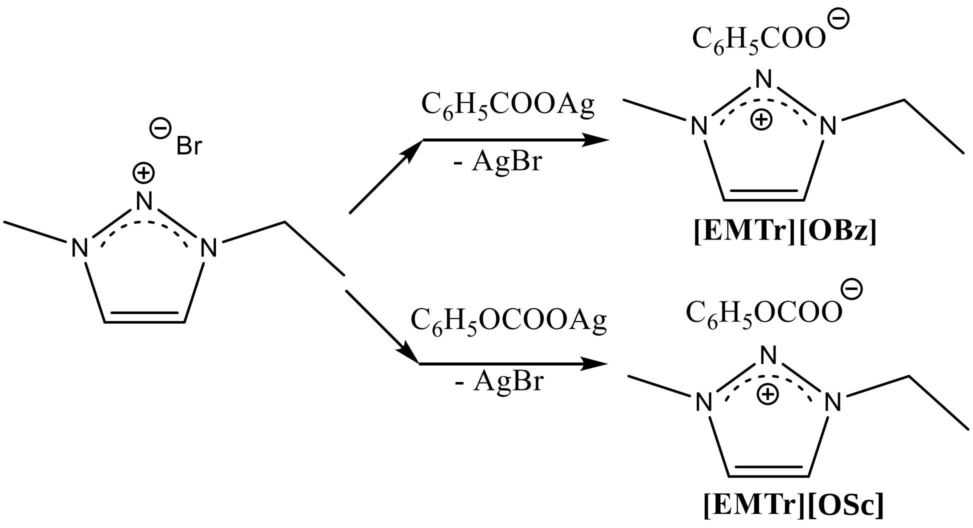

1-Ethyl-3-methyl-1,2,3-triazolium benzoate [EMTr][OBz]

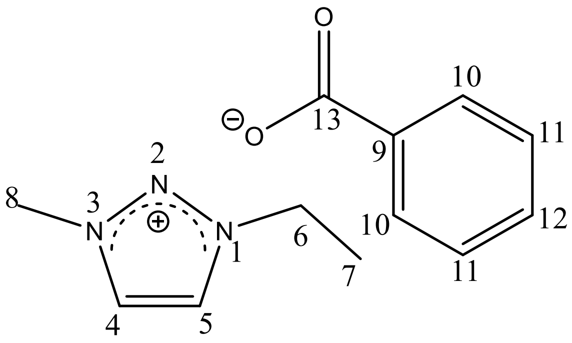

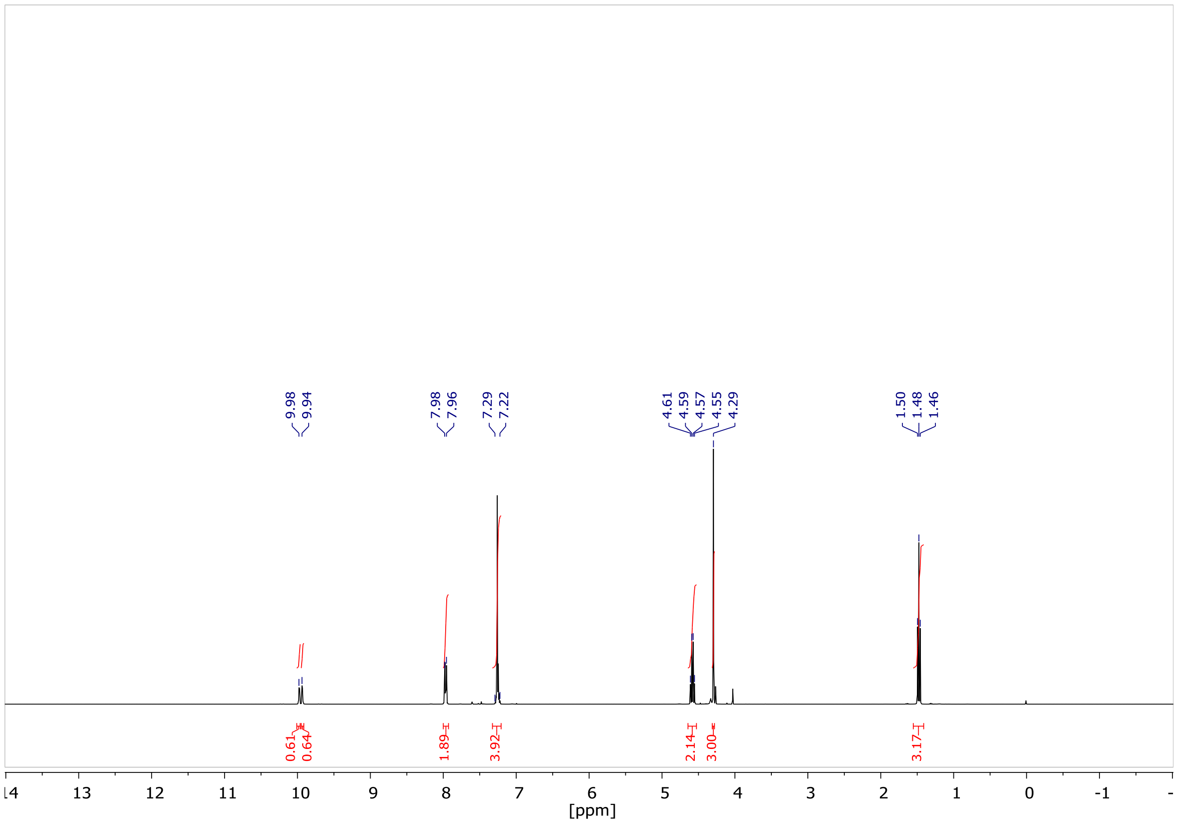

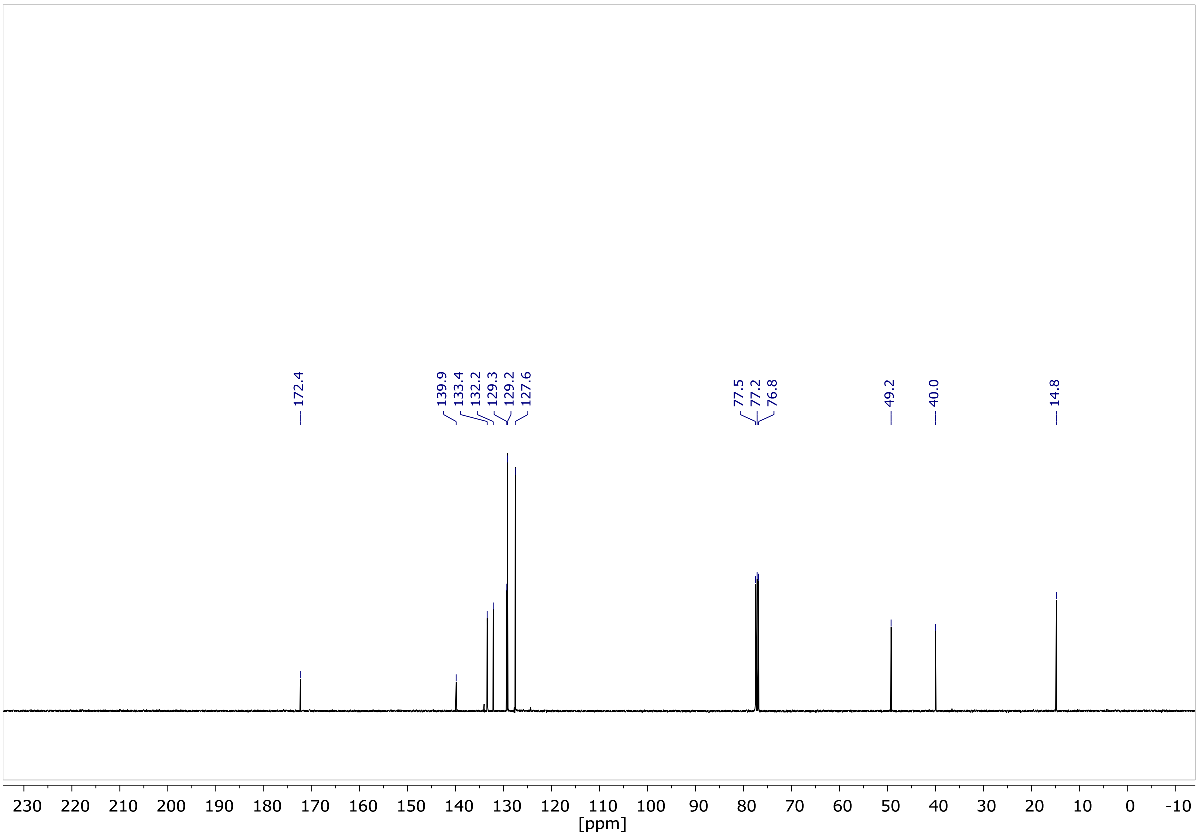

^{1}H NMR (400 MHz, $CDCl_3$, 27 °C): δ = 9.98 (*s*, 1H, H–4); 9.94 (*s*, 1H, H–5); 8.00 – 7.94 (*m*, 2H, H–11); 7.29 – 7.21 (*m*, 3H, H–10 + H–12); 4.58 (*q*, 2H, ^{3}J = 7.4 Hz, H–6); 4.29 (s, 3H, H–8); 1.48 (t, 3H, ^{3}J = 7.4 Hz, H–7) ppm;

^{13}C NMR (101 MHz, $CDCl_3$, 27 °C): δ = 172.4 (C–13); 139.9 (C–9);133.4 (C–11); 132.2 (C–12); 129.3 (C–10); 129.2 (C–4); 127.6 (C–5); 49.2 (C–6); 40.0 (C–8); 14.8 (C–7) ppm.

1-Ethyl-3-methyl-1,2,3-triazolium salicylate [EMTr][OSc]

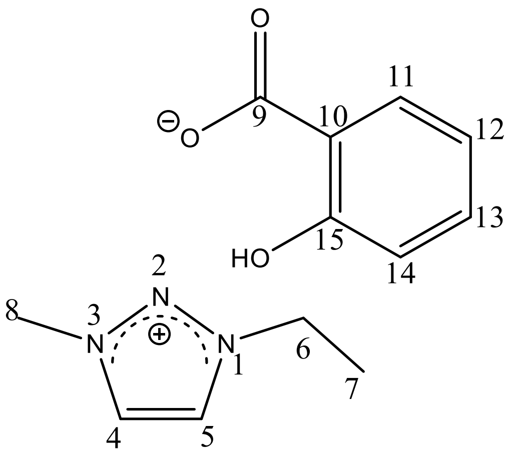

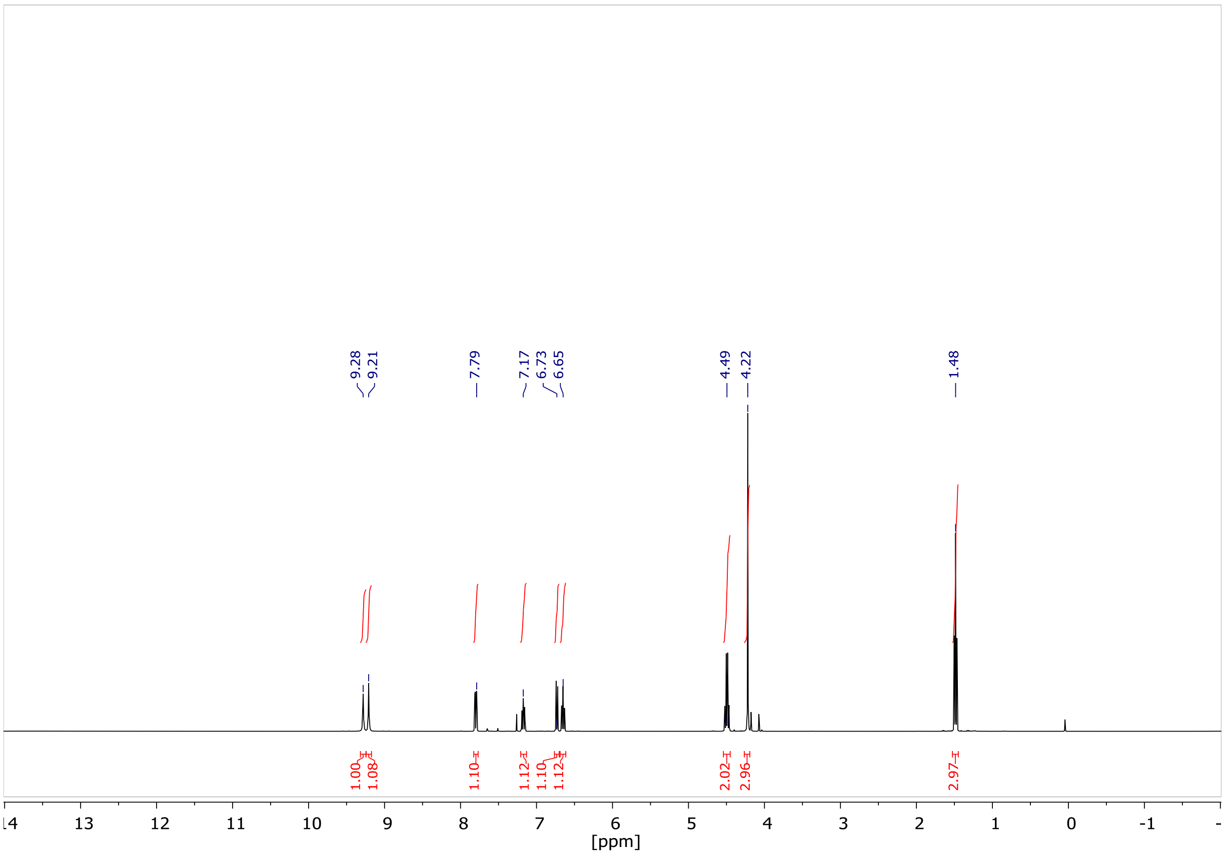

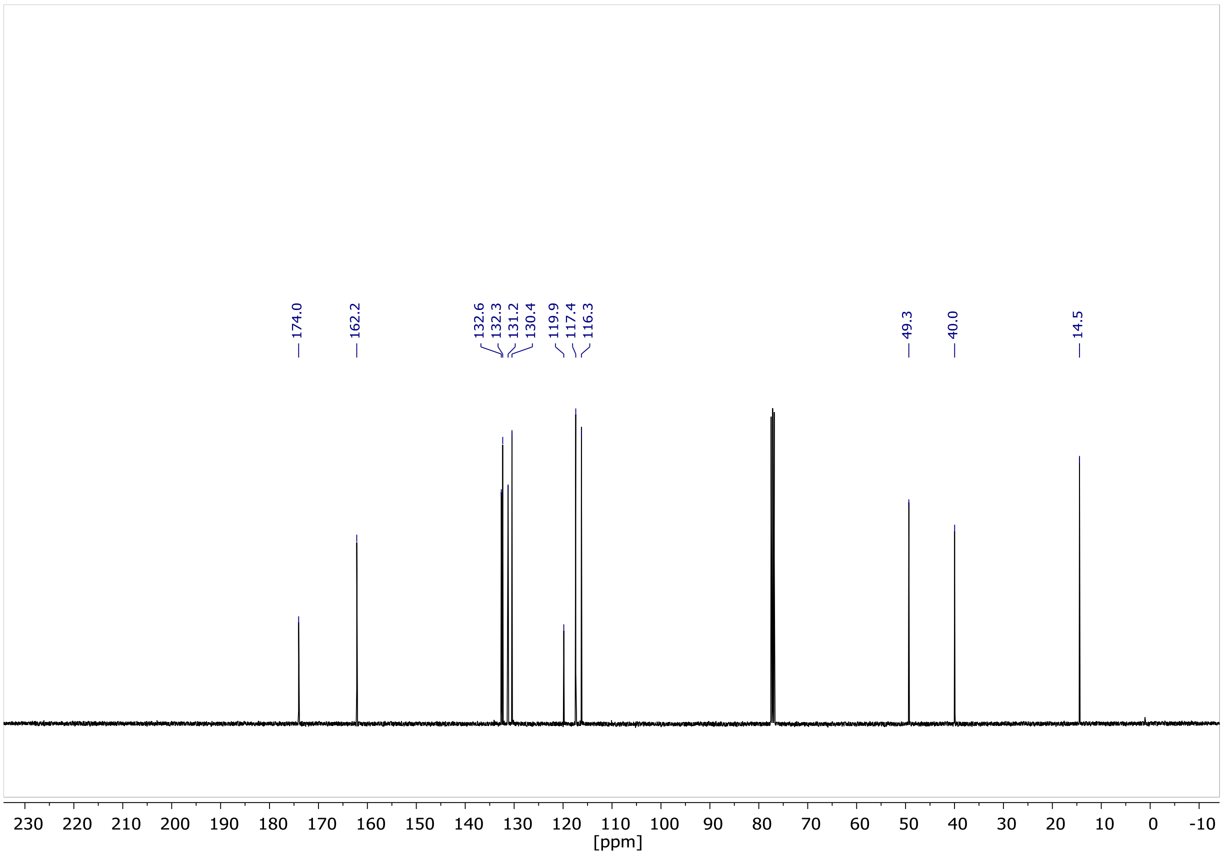

^{1}H NMR (400 MHz, $CDCl_3$, 27 °C): δ = 9.28 (*s*, 1H, H–4); 9.21 (*s*, 1H, H–5); 7.79 (*dd*, 1H, ^{3}J = 7.7 Hz, 1.8 Hz,); 7.17 (*ddd*, 1H, ^{3}J = 8.2, 7.2, 1.8 Hz,); 6.73 (*dd*, 1H, ^{3}J = 8.2, 1.1 Hz,); 6.65 (*td*, 1H, ^{3}J = 7.5, 1.2 Hz,); 4.49 (*q*, 2H, ^{3}J = 7.4 Hz, H–6); 4.22 (*s*, 3H, H–8); 1.48 (*t*, 3H, ^{3}J = 7.4 Hz, H–7) ppm;

^{13}C NMR (101 MHz, $CDCl_3$, 27 °C): δ = 174.0 (C–9); 162.2 (C–15); 132.6 (C–13); 132.3 (C–11); 131.2 (C–4); 130.4 (C–5); 119.9 (C–10); 117.4 (C–12); 116.3 (C–14); 49.3 (C–6); 40.0 (C–8); 14.5 (C–7) ppm.

Synthesis of imidazolium-based ionic liquids:

A solution of silver benzoate (20.46 mmol) in methanol (10 mL) was added to a solution of 1-ethyl-3-methylimidazolium chloride (20.46 mmol) in methanol (10 mL) and stirred under exclusion of light at room temperature for 10 min. The reaction mixture was filtrated and dried under vacuum.

To a solution of 1-ethyl-3-methylimidazolium chloride (20.46 mmol) in acetone (10 mL), a solution of silver salicylate (20.46 mmol) in acetone (10 mL) was added. The reaction was stirred for 10 min under exclusion of light at room temperature. After the reaction, the mixture was filtrated and dried.

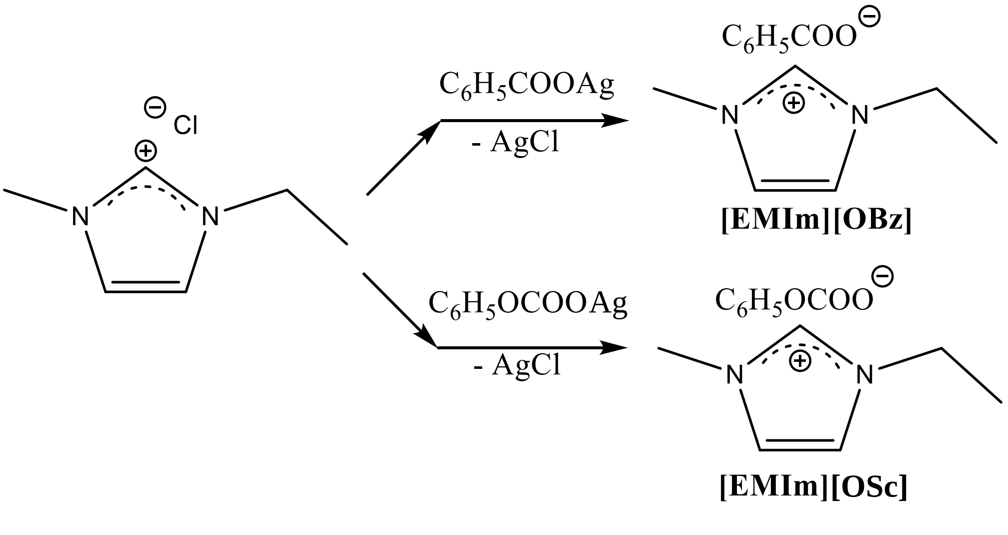

1-Ethyl-3-methylimidazolium benzoate [EMIm][OBz]

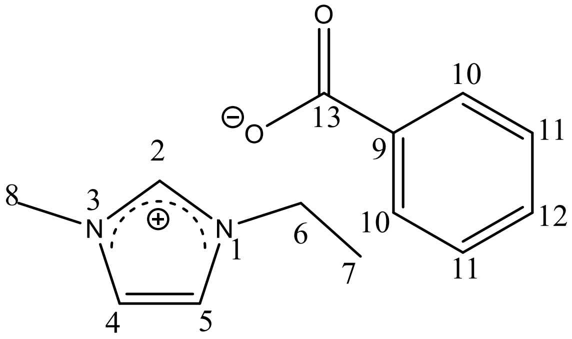

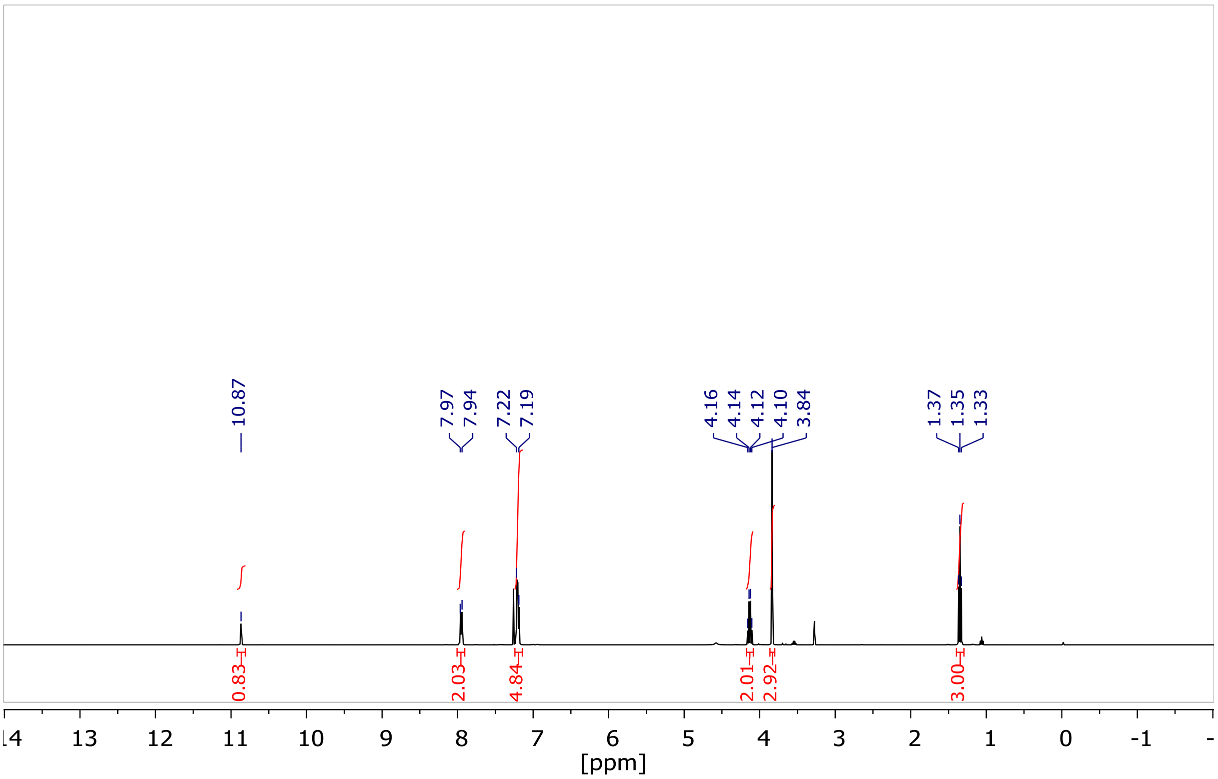

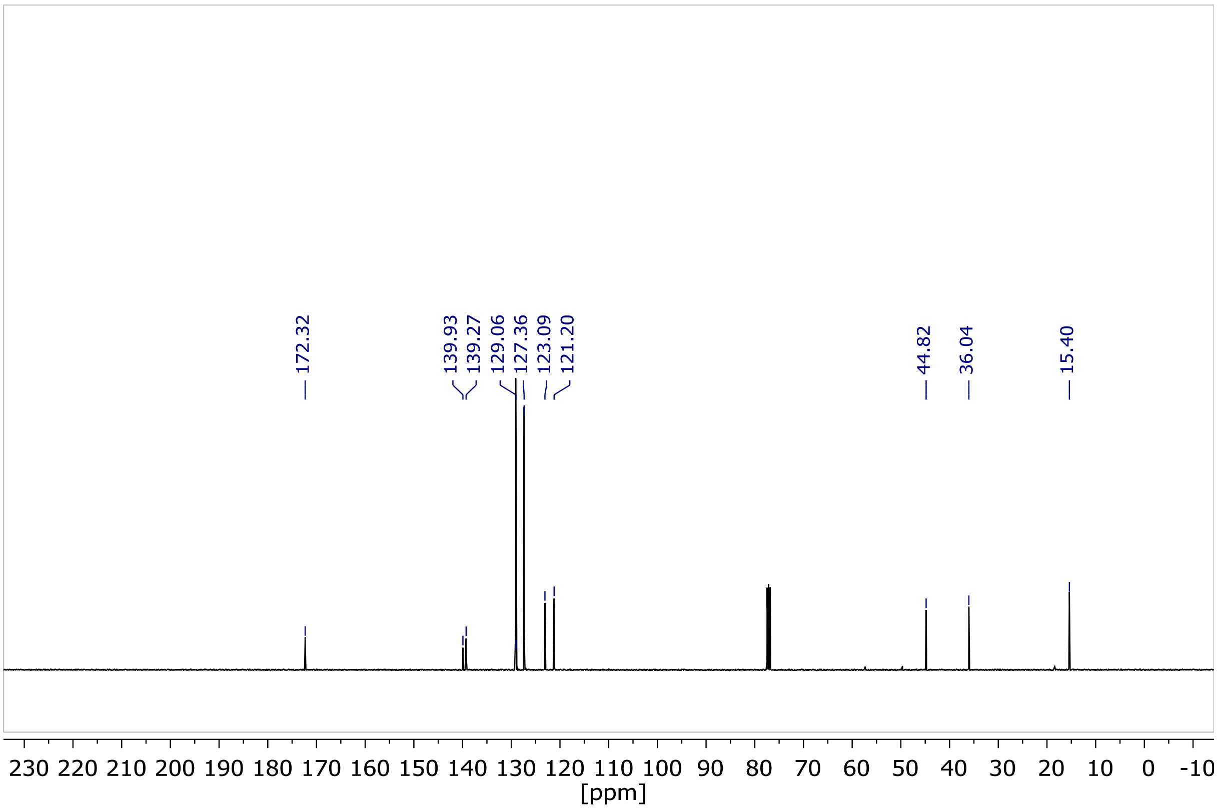

^{1}H NMR (400 MHz, $CDCl_3$, 27 °C): δ = 10.87 (*s*, 1H, H–2); 7.97 – 7.92 (*m*, 2H, H–4 + H–5); 7.23 – 7.17 (*m*, 5H, H–10 + H–11 + H–12); 4.13 (*q*, 2H, ^3J = 7.4 Hz, H–6); 3.84 (*s*, 3H, H–8); 1.35 (*t*, 3H, ^3J = 7.4 Hz, H–7) ppm;

^{13}C NMR (101 MHz, $CDCl_3$, 27 °C): δ = 172.3 (C–13); 139.9 (C–9);139.3 (C–2); 129.1 (C–11); 127.4 (C–12); 123.1 (C–10); 121.2 (C–4); 127.6 (C–5); 44.8 (C–6); 36.0 (C–8); 15.4 (C–7) ppm.

1-Ethyl-3-methylimidazolium salicylate [EMIm][OSc]

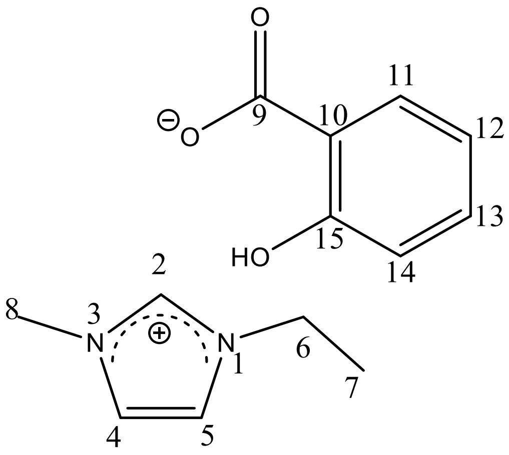

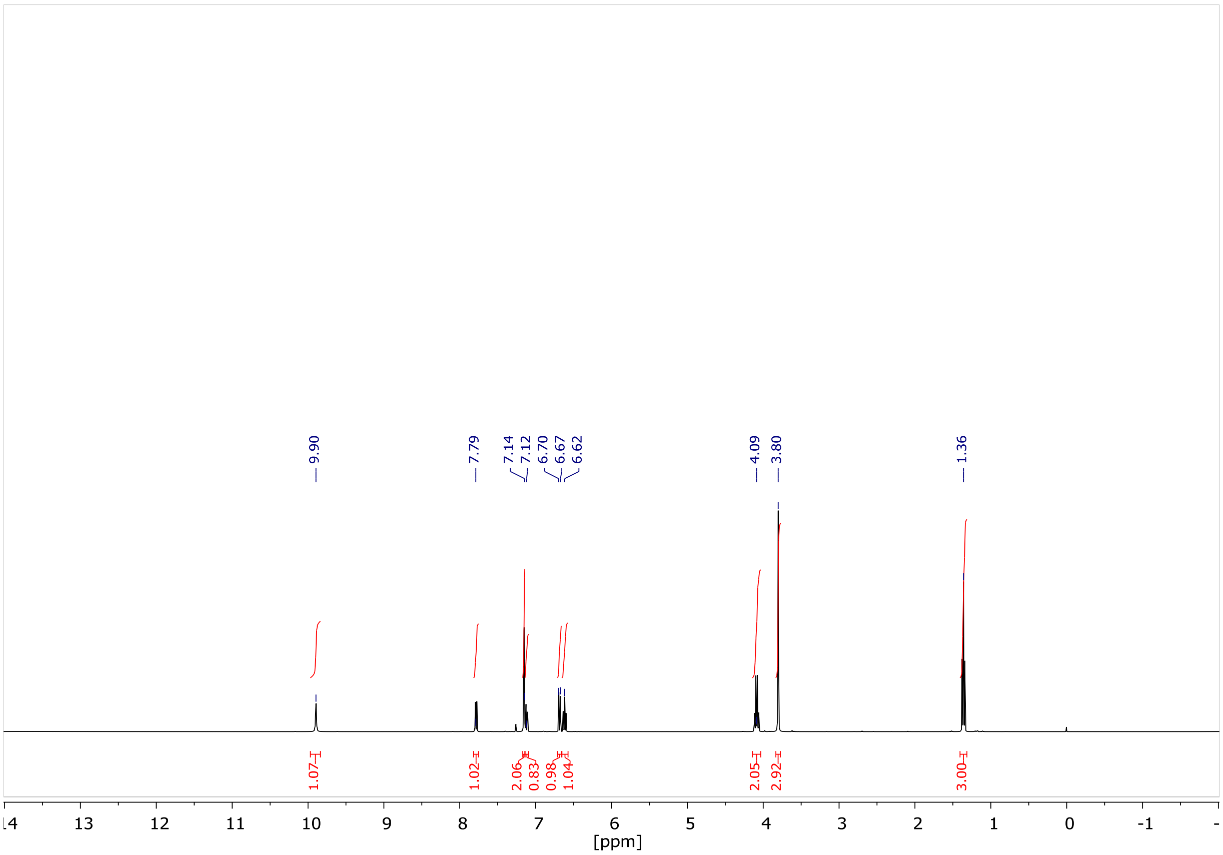

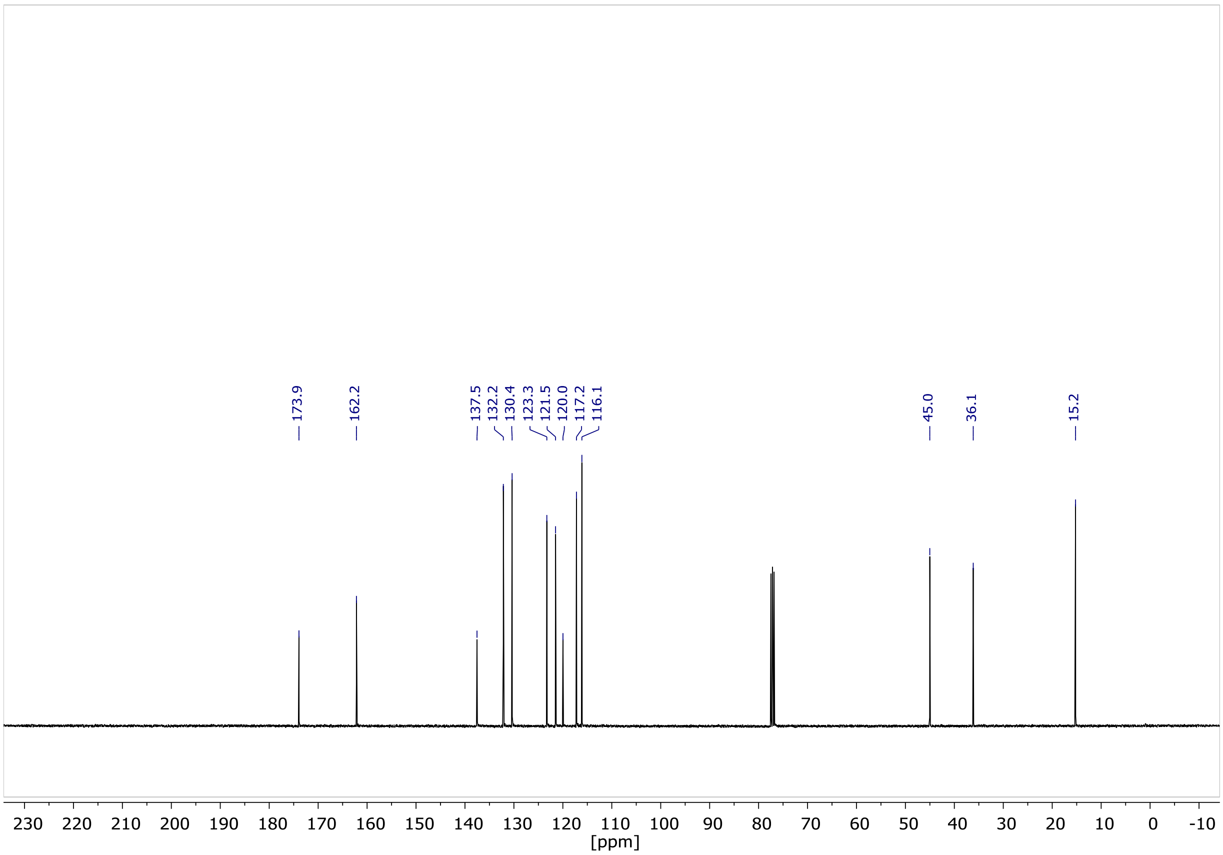

^{1}H NMR (400 MHz, $CDCl_3$, 27 °C): δ = 9.90 (*s*, 1H, H–2); 7.79 (*dd*, 1H, ^3J = 7.7 Hz, 1.8 Hz, H–11); 7.17 – 7.14 (*m*, 2H, H–4 + H–5); 7.13 – 7.10 (*m*, 1H, H–13); 6.71 – 6.66 (*m*, 1H, H–14); 6.65 (*td*, 1H, ^3J = 7.5, 1.2 Hz, H–12); 4.09 (*q*, 2H, ^3J = 7.4 Hz, H–6); 3.80 (*s*, 3H, H–8); 1.36 (*t*, 3H, ^3J = 7.4 Hz, H–7) ppm;

^{13}C NMR (101 MHz, $CDCl_3$, 27 °C): δ = 173.9 (C–9); 162.2 (C–15); 137.5 (C–2); 132.2 (C–13); 130.4 (C–11); 123.3 (C–4); 121.5 (C–5); 120.0 (C–10); 117.2 (C–12); 116.1 (C–14); 45.0 (C–6); 36.1 (C–8); 15.2 (C–7) ppm.

Viscosity:

Table S-1: Comparison of the viscosity from the four ILs with increasing temperature.

Temperature	Viscosity [Pa·s]			
[°C]	[EMIm][OBz]	[EMIm][OSc]	[EMTr][OBz]	[EMTr][OSc]
20	0.479 ± 0.03	0.495 ± 0.01	0.746 ± 0.01	1.388 ± 0.02
30	0.214 ± 0.01	0.216 ± 0.01	0.314 ± 0.01	0.528 ± 0.02
40	0.112 ± 0.01	0.111 ± 0.01	0.179 ± 0.05	0.241 ± 0.01
50	0.063 ± 0.01	0.065 ± 0.01	0.101 ± 0.02	0.125 ± 0.01
60	0.038 ± 0.02	0.040 ± 0.01	0.080 ± 0.06	0.075 ± 0.01
70	0.027 ± 0.01	0.025 ± 0.01	0.041 ± 0.02	0.047 ± 0.002
80	0.019 ± 0.00	0.022 ± 0.01	0.037 ± 0.02	0.032 ± 0.00
90	0.014 ± 0.00	0.017 ± 0.00	0.025 ± 0.01	0.024 ± 0.00

Density:

Table S-2: Comparison of the density measurement of the four ILs with increasing temperature.

Temperature	Density [g·cm^{-3}]			
[°C]	[EMIm][OBz]	[EMIm][OSc]	[EMTr][OBz]	[EMTr][OSc]
25	1.155	1.203	1.195	1.245
35	1.146	1.194	1.185	1.235
45	1.137	1.184	1.176	1.225
55	1.127	1.175	1.166	1.215
65	1.119	1.165	1.156	1.206
75	1.109	1.157	1.147	1.196
85	1.101	1.148	1.138	1.187

Water content and cellulose solubility in Ils:

Table S-3: Comparison of the water content and cellulose solubility of the four ILs.

Ionic Liquid	Water content [%]	Solubility [wt %]
[EMIm][OBz]	0.866	7.41
[EMIm][OSc]	0.331	2.89
[EMTr][OBz]	1.267	8.51
[EMTr][OSc]	0.649	4.77

Thermogravimetric Measurement

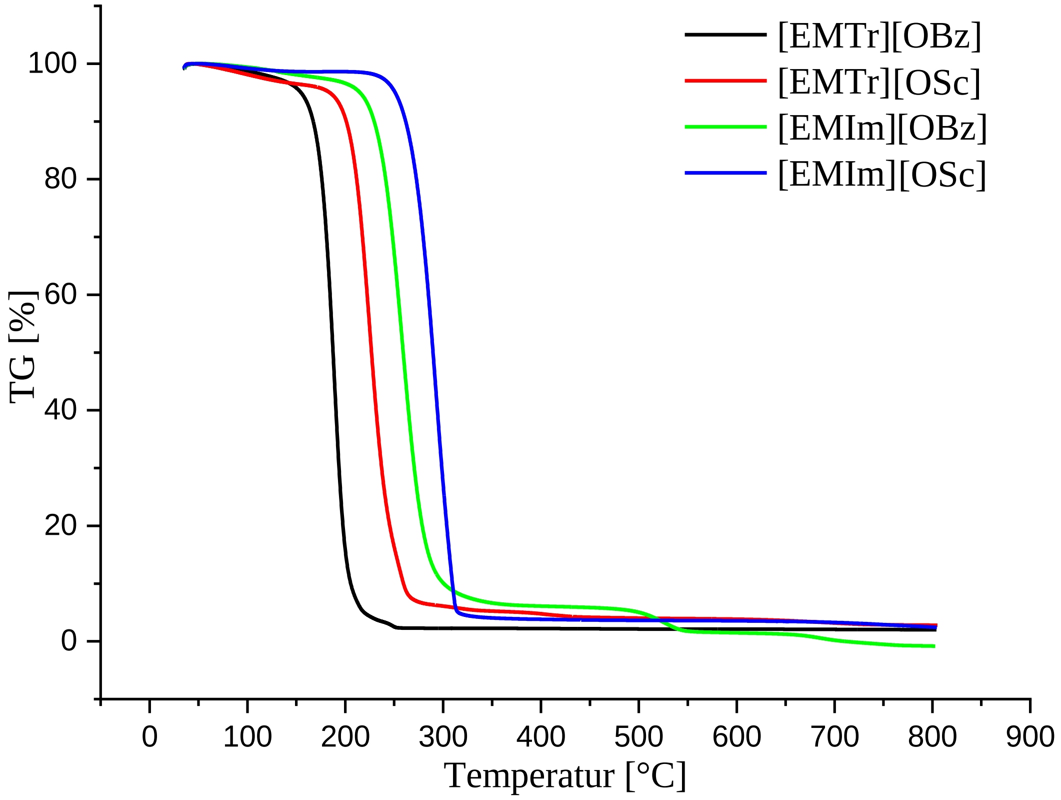

Figure S-1: The decomposition of the four ILs between 25°C and 800°C.

Computational Details

Partial charges for salicylate and benzoate anions:

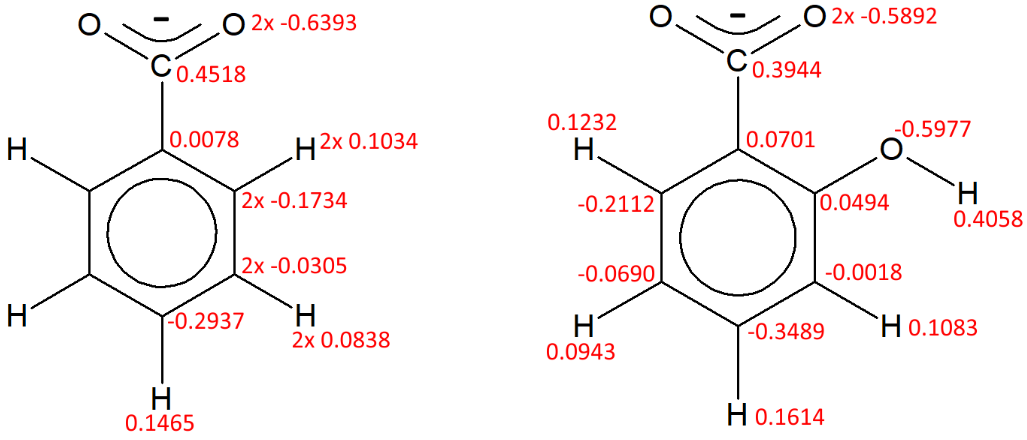

Figure S-2: Partial charges from RESP calculations in benzoate (left) and salicylate (right). For the simulations, these charges have been scaled down by a factor of 0.8.
